# Evaluation of the Quality of Evidence of the Association of Foods and Nutrients With Cardiovascular Disease and Diabetes: A Systematic Review

**DOI:** 10.1001/jamanetworkopen.2021.46705

**Published:** 2022-02-03

**Authors:** Victoria Miller, Renata Micha, Erin Choi, Dimitra Karageorgou, Patrick Webb, Dariush Mozaffarian

**Affiliations:** 1 Friedman School of Nutrition Science and Policy, Tufts University, Boston, Massachusetts; 2 Department of Food Science and Nutrition, University of Thessaly, Volos, Greece; 3 Department of Health Sciences, Northeastern University, Boston, Massachusetts

## Abstract

**Importance:**

Poor diet is a leading global factor associated with cardiometabolic disease (CMD). Understanding the quality of evidence of the associations between specific dietary factors and CMD, including effect size (relative risk [RR]) and uncertainty, is essential to guide policy and consumer actions to achieve healthy diet and public health goals.

**Objective:**

To assess the quality of evidence of the associations between specific dietary factors and CMD as well as the quantitative evidence for RRs and the uncertainty of these risk estimates.

**Evidence Review:**

PubMed and the reference lists of eligible articles were searched between May 1, 2015, and February 26, 2021, for systematic reviews with meta-analyses of randomized clinical trials and prospective cohort studies that analyzed the consumption of 1 or more of the dietary factors of interest; reported dose-response meta-analyses; included healthy adults; and assessed 1 or more of the outcomes of interest. Study characteristics and RR estimates were extracted in duplicate. For identified associations, quality of evidence was assessed using the Bradford-Hill criteria for causation.

**Findings:**

A total of 2058 potentially relevant reports were identified, from which 285 full-text articles were assessed for eligibility. The final selection of articles included 28 meta-analyses representing 62 associations between diet and CMD. Among these associations, 10 foods, 3 beverages, and 12 nutrients had at least probable evidence of associations with coronary heart disease, stroke, and/or diabetes. Most RRs ranged from 0.87 to 0.96 per daily serving change for protective associations and from 1.06 to 1.15 per daily serving change for harmful associations. Most identified associations were protective (n = 38) and a smaller number were harmful (n = 24), with a higher risk associated with higher intake.

**Conclusions and Relevance:**

This systematic review summarized the current quality of evidence of the associations of specific dietary factors with coronary heart disease, stroke, and diabetes. These findings may inform dietary guidance, the assessment of disease burden in specific populations, policy setting, and future research.

## Key Points

**Question:**

What is the quality of evidence for the association of foods, beverages, and nutrients with cardiometabolic outcomes?

**Findings:**

In this systematic review of 28 dose-response meta-analyses representing 62 associations between diet and disease, 10 foods, 3 beverages, and 12 nutrients had at least probable evidence of their associations with coronary heart disease, stroke, and/or diabetes.

**Meaning:**

These findings describe the current quality of evidence of the associations between dietary factors and cardiometabolic disease, which may inform dietary guidance, assessment of disease burden, policy setting, and future research.

## Teaser

This systematic review examines meta-analyses of randomized clinical trials and cohort studies for the magnitude of effect of foods, beverages, and nutrients for cardiometabolic disease.

## Introduction

Global rates of cardiometabolic disease (CMD), including cardiovascular disease (CVD) and type 2 diabetes, have steadily increased over the past 3 decades.^1,2^ In 2019, an estimated 523 million adults were living with CVD and 463 million adults were living with diabetes worldwide.^1,3^ Poor diet is a leading modifiable risk factor for CMD,^4^ with millions of deaths estimated to be attributable to low intake of healthy foods and high intake of unhealthy foods.^4,5,6,7,8,9,10^ The association between diet and CMD has been a major focus of health sciences research since the 1950s.^11^ The evidence used today is based on a range of paradigms, including findings from prospective observational cohort studies, randomized clinical trials (RCTs) of clinical risk factors, RCTs of clinical end points, and supportive experimental studies, such as those in animals.^12^ Results from this diversity of scientific approaches with varying strengths and limitations may be evaluated for specific evidence of associations between diet and disease as well as for the best evidence available.

Previous work that assessed the quality of evidence of various dietary risk factors for CMD identified 10 foods and 7 nutrients with probable or convincing evidence of associations between diet and cardiovascular outcome.^13^ In the 6 years since the last evidence review was completed (May 1, 2015), many new scientific studies of diet and CMD have been published. Furthermore, the earlier review did not include many key foods and nutrients (eg, potatoes, dietary protein, and subtypes of dietary fiber), several of which may have an important role in CMD. A number of umbrella reviews have reported compiled information from meta-analyses of observational studies on dietary factors and CMD outcomes, but they did not assess the quality of evidence of these associations.^14,15,16,17,18,19,20,21,22,23,24,25,26,27,28,29,30,31,32,33,34^ Thus, the latest evidence of the associations of most dietary factors with CMD is not well established.

To address this gap in the literature, we conducted a systematic review of the literature. We aimed to assess the quality of evidence for the associations between specific dietary factors and CMD as well as the quantitative evidence for effect sizes (relative risks [RRs]) and the uncertainty of these estimates.

## Methods

### Quality of Evidence

Using previously established methods for evaluating the evidence of associations between diet and CMD,^13,35^ we selected the following outcomes of interest: incident total or fatal CVD, coronary heart disease (CHD), myocardial infarction, stroke, ischemic stroke, hemorrhagic stroke, and diabetes. Briefly, for each association between diet and CMD, 2 of us (V.M. and D.M.) independently and in duplicate graded the quality of evidence according to the Bradford-Hill criteria for causation: strength, consistency, temporality, coherence, specificity, analogy, plausibility, biological gradient, and experiment.^36^ Detailed descriptions of these criteria are provided in eAppendix 1 in the Supplement. We examined the associations between diet and CMD with probable or convincing evidence of associations, and the evidence for many of these associations was appraised as not meeting the Bradford-Hill criteria. We also qualitatively assessed the concordance of our assessments with the grading criteria of the World Health Organization^37^ and the World Cancer Research Fund/American Institute for Cancer Research.^38^ We focused on foods, beverages, and nutrients and did not assess dietary supplements or alcohol use.

### Literature Searches for Associations

For this systematic review, we followed the Preferred Reporting Items for Systematic Reviews and Meta-analyses (PRISMA) reporting guideline. Between May 1, 2015, and February 26, 2021, we searched PubMed for systematic reviews with meta-analyses of RCTs and prospective cohort studies using standardized search terms (details on the search method, search terms, and search results are provided in the eMethods, eAppendix 2, and eTable 1 in the Supplement). In addition, we reviewed the reference lists from all of the retrieved full-text articles for additional relevant studies. One of us (V.M.) conducted the search and the title and abstract screening, and 3 of us (V.M., E.C., and D.K.) independently and in duplicate screened relevant full-text articles. Disagreements were resolved by consensus and with another investigator (D.M.).

Data on participant race and ethnicity were not collected except in RCTs of the association between sodium and systolic blood pressure (SBP). The RRs are reported among Black participants.

### Inclusion and Exclusion Criteria

Informed by previous studies and expert knowledge, we focused on 43 food, beverage, or nutrient groups of interest: fruits, fruit juices, vegetables, potatoes, beans or legumes, nuts or seeds, whole grains, refined grains, milk, yogurt, cheese, unprocessed red meats, processed meats, fish or seafood, lean fish, fatty fish, eggs, sugar-sweetened beverages (SSBs), non-nutritive sweetened beverages, coffee, tea, chocolate (cocoa), saturated fatty acids, monounsaturated fatty acids, polyunsaturated fatty acids, seafood omega-3 fatty acids, plant omega-3 fatty acids, trans-fatty acids, protein, animal protein, plant protein, dietary cholesterol, dietary fiber, cereal fiber, fruit fiber, vegetable fiber, legume fiber, glycemic index, glycemic load, dietary sodium, dietary potassium, dietary calcium, and total energy. We did not separately assess low-fat and whole-fat subtypes of dairy given the mixed evidence on the harms vs benefits of dairy fat.^39,40,41^

Studies were included if they met each of the following criteria: (1) systematic review with meta-analysis of RCTs and prospective cohort studies (including nested case-control design); (2) analyzed intake of 1 or more of the food, beverage, or nutrient groups of interest; (3) reported dose-response meta-analyses using all available data as opposed to only comparisons of high- and low intake category; (4) included healthy adults aged 18 years or older; and (5) assessed 1 or more of the CMD outcomes of interest. For sodium, we also reviewed studies on SBP and diastolic blood pressure. For SSBs and non-nutritive sweetened beverages, we included studies on changes in intake and overweight or obesity. Given the intersection of CVD risk among people with diabetes, we also selected meta-analyses of the associations of dietary exposures with CVD end points among people with diabetes. When more than 1 meta-analyses were identified for any association between diet and CMD, we included the meta-analysis with the greatest number of studies and events. We excluded meta-analyses that assessed only fatal CHD, fatal myocardial infarction, or fatal stroke, including only retrospective case-control or cross-sectional studies; reported crude RR estimates, including fewer than 3 individual studies in the dose-response analysis; or reported only nonparametric associations. The complete inclusion and exclusion criteria are described in eAppendix 2 in the Supplement.

### Data Extraction

For each included meta-analysis, 2 reviewers (V.M. and E.C.) independently and in duplicate extracted the following characteristics using a standardized electronic spreadsheet: name of first author, year of publication, study name, study design, literature search date, databases searched, dietary exposure (definition, assessment method, and dose), outcome (definition and ascertainment method), inclusion and exclusion criteria, population, number of included studies and cohorts, length of follow-up, sample size, number of cases, analysis method, RR estimates with corresponding 95% CIs, and covariates. If the original meta-analysis did not report all required data but cited the included individual studies, we extracted the data from the individual studies. For each association between diet and CMD, we standardized the risk estimates and corresponding uncertainty to an established serving size.

## Results

### Dietary Factors With Probable or Convincing Evidence

**Figure 1.**
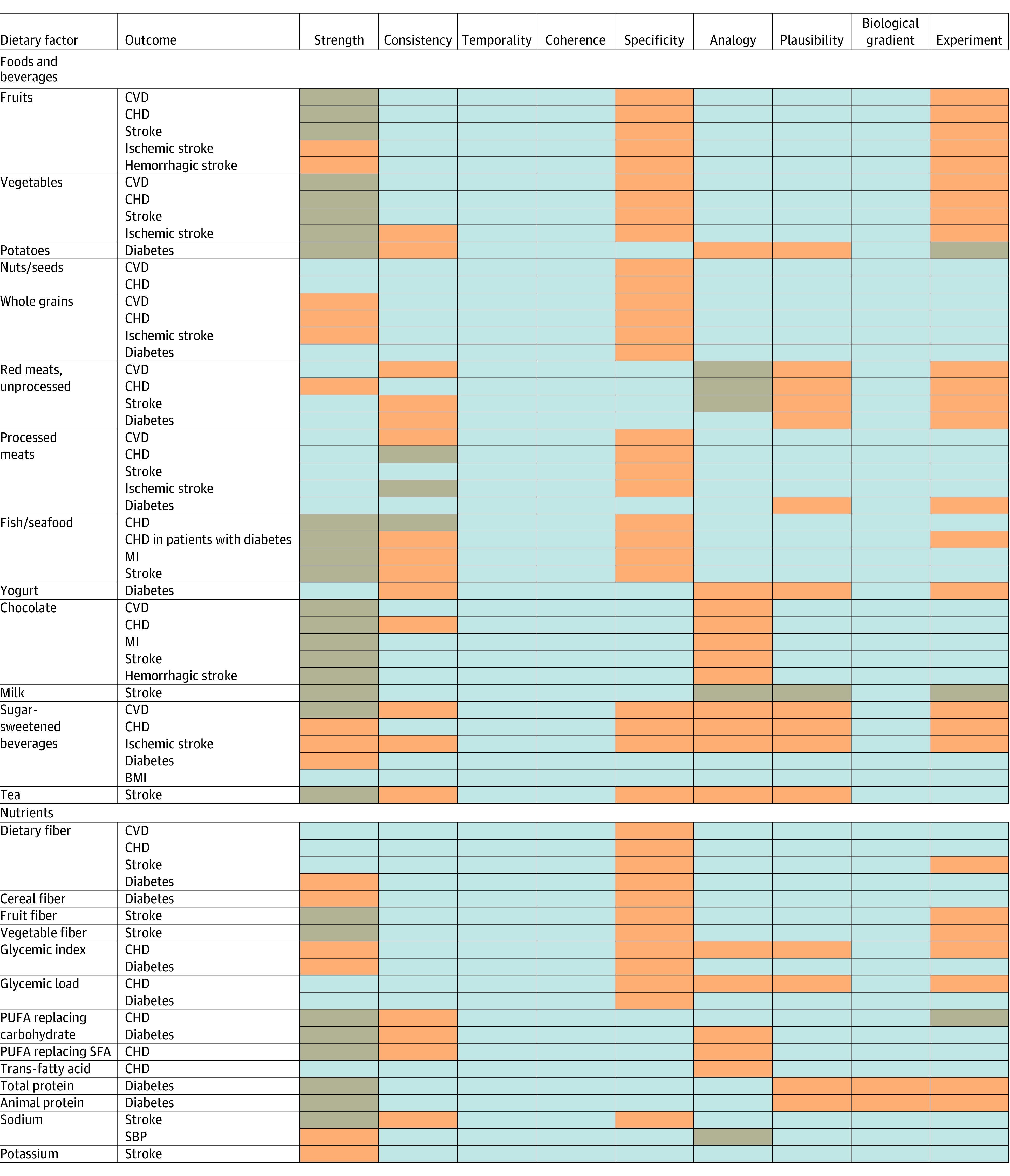
Grading of Evidence of the Associations of Specific Dietary Factors With Cardiometabolic Outcomes The 9 Bradford-Hill criteria for grading the evidence were strength, consistency, temporality, coherence, specificity, analogy, plausibility, biological gradient, and experiment. Each criterion is defined in eAppendix 1 in the Supplement. Gray indicates consistent evidence from well-designed studies with relatively few limitations; orange, consistent evidence from several well-designed studies with some important limitations; and brown, emerging evidence from a few studies or conflicting results from several studies. BMI indicates body mass index (calculated as weight in kilograms divided by height in meters squared); CHD, coronary heart disease; CVD, cardiovascular disease; MI, myocardial infarction; PUFA: polyunsaturated fatty acid; SBP, systolic blood pressure; and SFA, saturated fatty acid.

This systematic search identified 2058 potentially relevant reports, from which 285 full-text articles were retrieved through title and abstract screening (eFigure 1 in the Supplement). The final selection of articles included 28 meta-analyses^42,43,44,45,46,47,48,49,50,51,52,53^ representing 62 associations between diet and CMD. Among these associations, 25 dietary factors (10 foods, 3 beverages, and 12 nutrients) were identified that had probable or convincing evidence (which was graded using the Bradford-Hill criteria) of their associations with specific CMD outcomes (Table; Figure 1). Most identified associations were protective (n = 38), and a smaller number of associations were harmful (n = 24), with a higher risk associated with higher intake.

**Table.** Dietary Factors and Cardiometabolic Outcomes With Probable or Convincing Evidence of Associations^a^

Dietary factor	Cardiovascular outcome	Metabolic outcome
Protective association		
Fruits^b^	CVD, CHD, stroke, ischemic stroke, hemorrhagic stroke	NA
Vegetables^c^	CVD, CHD, stroke, ischemic stroke	NA
Nuts or seeds	CVD, CHD	NA
Whole grains	CVD, CHD, ischemic stroke	Diabetes
Fish or seafood^d^	CHD, CHD in patients with diabetes, MI, stroke	NA
Yogurt	NA	Diabetes
Chocolate	CVD, CHD, MI, stroke, hemorrhagic stroke	NA
Milk	Stroke	NA
Tea	Stroke	NA
Dietary fiber	CVD, CHD, stroke	Diabetes
Cereal fiber	NA	Diabetes
Fruit fiber	Stroke	NA
Vegetable fiber	Stroke	NA
PUFA replacing carbohydrate	CHD	Diabetes
PUFA replacing SFA	CHD	NA
Potassium	Stroke	NA
Harmful association		
Potatoes	NA	Diabetes
Red meats, unprocessed^e^	CVD, CHD, stroke	Diabetes
Processed meats^f^	CVD, CHD, stroke, ischemic stroke	Diabetes
SSBs^g^	CVD, CHD, ischemic stroke	Diabetes, high BMI
Glycemic index	CHD	Diabetes
Glycemic load	CHD	Diabetes
Trans-fatty acid	CVD	NA
Total protein	NA	Diabetes
Animal protein	NA	Diabetes
Sodium	Stroke, SBP	NA

Abbreviations: BMI, body mass index (calculated as weight in kilograms divided by height in meters squared); CHD, coronary heart disease; CVD, cardiovascular disease; MI, myocardial infarction; NA, not applicable; PUFA: polyunsaturated fatty acid; SBP, systolic blood pressure; SFA, saturated fatty acid; SSB, sugar-sweetened beverage.

^a^ eAppendix 1 in the Supplement provides details on the Bradford-Hill criteria for grading the evidence of each association.

^b^ Excluding 100% juices.

^c^ Excluding vegetable juices; starchy vegetables, such as potatoes and corn; and salted or pickled vegetables. Because certain beans or legumes (eg, black beans and lentils) were commonly included as vegetables in many of the identified studies, the associations identified for vegetables should be considered as representing the outcome of vegetables, including beans or legumes. Associations of beans or legumes were also separately evaluated.

^d^ A previous review^13^ found evidence of associations of dietary seafood omega-3 fatty acids with fatal CHD^54^ but not total or nonfatal CHD, and the former was excluded from the outcomes of this study.

^e^ Beef, lamb, or pork; excluding poultry, fish, eggs, and processed meats.

^f^ Any meat preserved by smoking, curing, salting, or addition of chemical preservatives, such as bacon, salami, sausages, hot dogs, or processed deli or luncheon meats, excluding fish and eggs.

^g^ In addition to the association of SSBs with adiposity (obesity), evidence from prospective studies suggested an additional, BMI-independent association of SSBs with incidence of type 2 diabetes, CVD, CHD, and MI.

We did not find sufficient evidence of associations for 18 dietary factors, including fruit juice, beans or legumes, refined grains, cheese, lean fish, fatty fish, eggs, non-nutritive sweetened beverages, coffee, saturated fatty acids, monounsaturated fatty acids, dietary seafood or plant omega-3 fatty acids, plant protein, dietary cholesterol, legume fiber, dietary calcium, and total energy. For many of these dietary factors, the meta-analyses of observational studies identified associations with CMD outcomes, but they were based on too few studies (≤2) and/or did not meet 1 or more of the Bradford-Hill criteria (eTable 3 in the Supplement).

### Quality of Evidence of Associations for CVD

**Figure 2.**
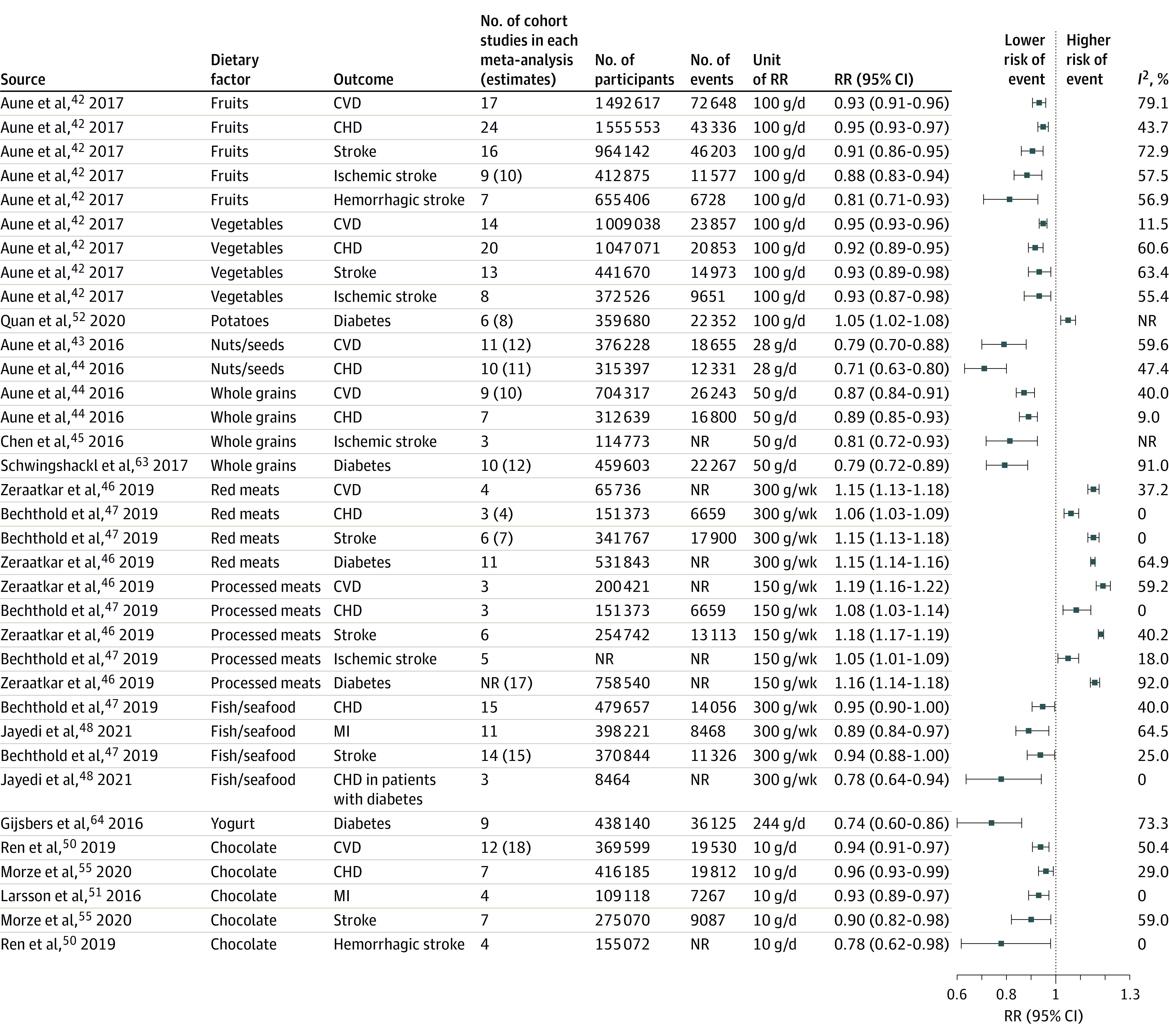
Relative Risks (RRs) of the Associations of Foods With Risk of Cardiovascular Diseases (CVDs) and Type 2 Diabetes Boxes in the plot show the RRs from the meta-analyses, and the horizontal lines through the boxes show the length of the 95% CIs. Associations supported by fewer than 3 studies that did not reference the individual studies were excluded. The number of RR estimates can be higher than the number of studies if there were more than 1 group in a randomized clinical trial, if estimates were separated by age or sex in prospective cohort studies, or if more than 1 prospective cohort study was included in a single study. Upper 95% CIs for association of fish or seafood with coronary heart disease (CHD) and stroke are significant (<1.0) when 3 significant digits are reported. MI indicates myocardial infarction; NR, not reported.

**Figure 3.**
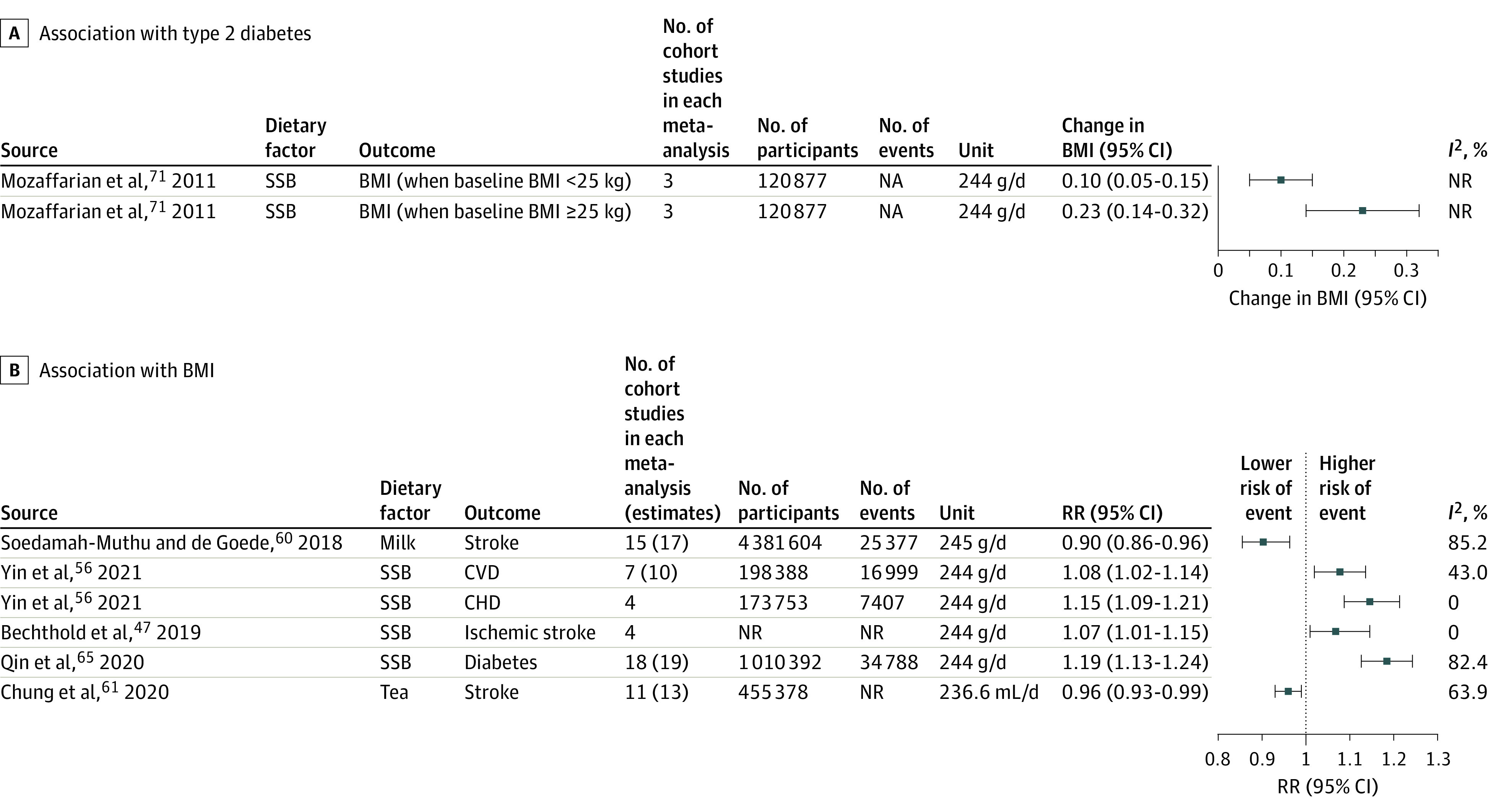
Relative Risks (RRs) of the Associations of Beverages With Risk of Cardiovascular Diseases (CVDs) and Type 2 Diabetes and With Body Mass Index (BMI) Boxes in the plot show the change in BMI (calculated as weight in kilograms divided by height in meters squared; A) and the RRs from the meta-analyses (B). The horizontal lines through the boxes show the length of the 95% CIs. Associations supported by fewer than 3 studies that did not reference the individual studies were excluded. The number of RR estimates can be higher than the number of studies if there were more than 1 group in a randomized clinical trial, if estimates were separated by age or sex in prospective cohort studies, or if more than 1 prospective cohort study was included in a single study. BMI indicates body mass index; CHD, coronary heart disease; NA, not applicable; NR, not reported; and SSB, sugar-sweetened beverage.

**Figure 4.**
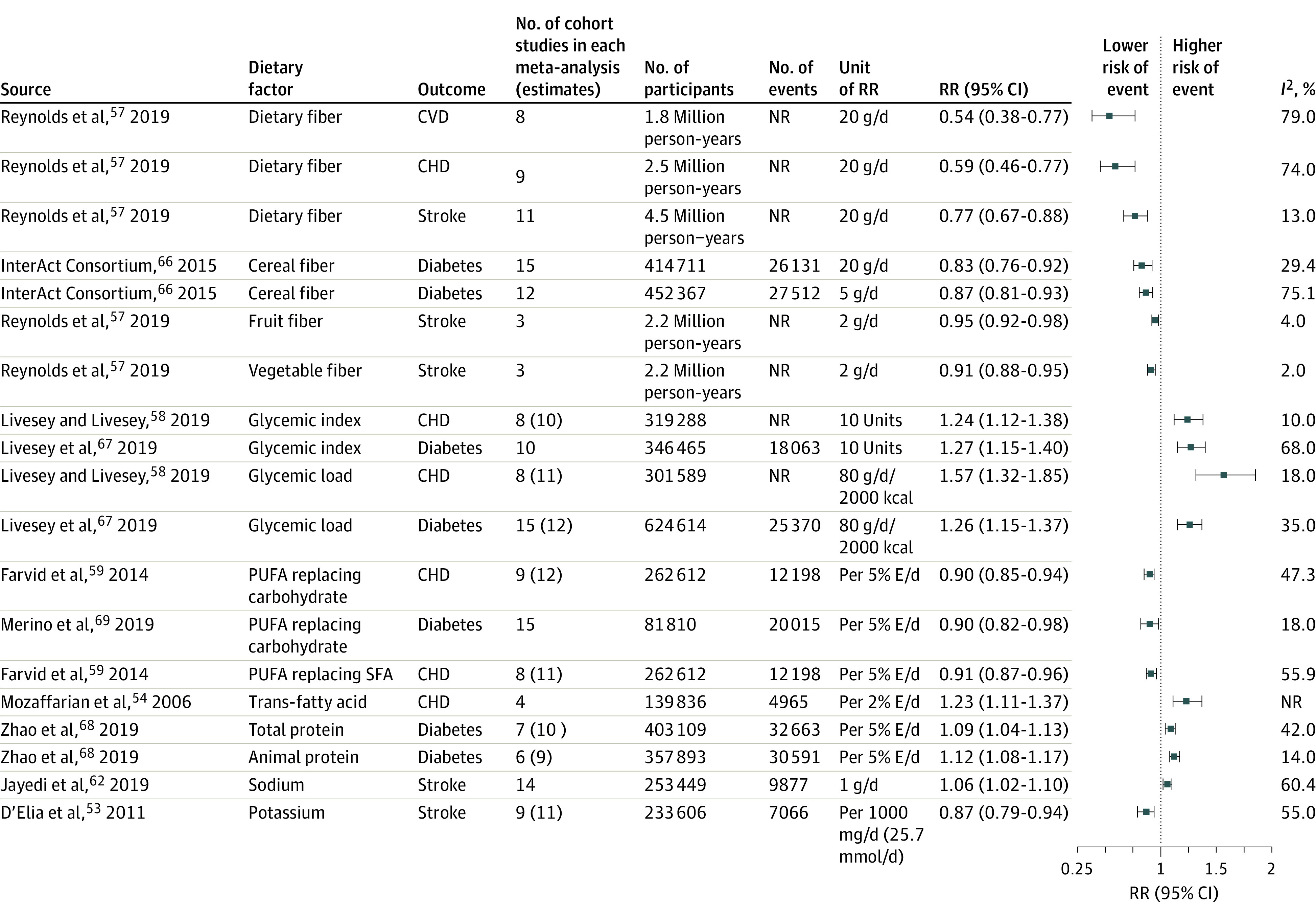
Relative Risks (RRs) of the Associations of Nutrients With Risk of Cardiovascular Diseases (CVDs) and Type 2 Diabetes Boxes in the plot show the RRs from the meta-analyses, and the horizontal lines through the boxes show the length of the 95% CIs. Associations supported by fewer than 3 studies that did not reference the individual studies were excluded. Number of RR estimates can be higher than the number of studies if there were more than 1 group in a randomized clinical trial, if estimates were separated by age or sex in prospective cohort studies, or if more than 1 prospective cohort study was included in a single study. CHD indicates coronary heart disease; NR, not reported; PUFA, polyunsaturated fatty acid; and SFA, saturated fatty acid.

A total of 21 dietary factors had probable or convincing evidence of associations for different CVD end points^42,43,44,45,46,47,48,49,50,51^ (Figure 2, Figure 3, and Figure 4; eFigure 2 in the Supplement). Among all CVD outcomes, fruits, vegetables, chocolate, processed meats, fish or seafood, and SSBs had the greatest number of identified associations. The most frequently reported associations were for CHD (14 associations),^42,44,47,54,55,56,57,58,59^ stroke (13 associations),^42,46,47,53,55,57,60,61,62^ and total CVD (10 associations).^42,44,46,50,52,56,57^ The associations with the largest number of published individual research were between sodium and SBP (103 RCTs),^5^ fruits and CHD (24 cohort studies),^42^ and vegetables and CHD (20 cohort studies).^42^ Associations between whole grains and ischemic stroke,^45^ unprocessed red meat and CHD,^47^ processed meat and CVD,^46^ processed meat and CHD,^46^ fish or seafood and CHD in patients with diabetes,^48^ fruit fiber and stroke,^57^ and vegetable fiber and stroke^57^ had the fewest number of published articles, with 3 cohort studies each.

The number of participants in each meta-analysis ranged from approximately 7000 in RCTs of the association between sodium and SBP to approximately 1.5 million in cohort studies of the association between fruits and CHD. The greatest number of events was for the association between fruits and CVD (72 648 events), and the fewest number of events was for the association between trans-fatty acid and CHD (4965 events). Most RRs ranged from 0.87 to 0.96 per daily serving change for protective associations and from 1.06 to 1.15 per daily serving change for harmful associations. Larger beneficial associations were found between fiber and CVD (RR, 0.54; 95% CI, 0.38-0.77 per 20 g/d),^57^ fiber and CHD (RR, 0.59; 95% CI, 0.46-0.77 per 20 g/d),^57^ and nuts or seeds and CHD (RR, 0.71; 95% CI, 0.63-0.80 per 28 g/d)^44^ (Figure 2 and Figure 4) (eTable 2 in the Supplement shows the units and RRs reported in each meta-analysis). The most harmful associations were observed between glycemic load and CHD (RR, 1.57; 95% CI, 1.32-1.85 per 80 g/d/2000 kcal),^58^ glycemic index and CHD (RR, 1.24; 95% CI, 1.12-1.38 per 10 units),^58^ and trans-fatty acids and CHD (RR, 1.23; 95% CI, 1.11-1.37 per 2% E/d)^54^ (Figure 4).

Compared with the 2015 search and quality-of-evidence analysis,^13,53,54,55,56,57,58,59,60,61,62^ the present systematic review identified 7 additional dietary factors with at least probable evidence of associations for CVD. These dietary factors were unprocessed red meats, chocolate, milk, tea, fruit fiber, vegetable fiber, and glycemic index.

### Quality of Evidence of Associations for Diabetes

Thirteen dietary factors were identified with at least probable evidence of associations for diabetes^46,52,63,64,65,66,67,68,69^ (Figure 2, Figure 3, and Figure 4). The number of reported cohort studies ranged from 6 to 18, with the total number of participants ranging from approximately 82 000 (for polyunsaturated fatty acid replacing carbohydrate) to approximately 1 million (for SSBs) and the total number of incident diabetes cases ranging from 18 063 (for glycemic index) to 36 125 (for yogurt).

Five dietary factors (whole grains, yogurt, fiber, cereal fiber, and polyunsaturated fatty acid replacing carbohydrate) had protective associations, whereas 8 (potatoes, unprocessed red meat, processed meat, SSBs, glycemic index, glycemic load, protein, and animal protein) had harmful associations with diabetes. The protective association was largest for yogurt (RR, 0.74; 95% CI, 0.60-0.86 per 244 g/d),^64^ and other protective associations ranged from 0.70 to 0.90. Glycemic index (RR, 1.27; 95% CI, 1.15-1.40 per 10 units),^67^ glycemic load (RR, 1.26; 95% CI, 1.15-1.37 per 80 g/d/2000 kcal),^67^ and SSBs (RR, 1.19; 95% CI, 1.13-1.24 per 244 g/d)^65^ were estimated to be the most harmful associations (Figure 2, Figure 3, and Figure 4).

Compared with the 2015 search and quality-of-evidence analysis,^13^ the present review identified new dietary factors associated with diabetes: potatoes, cereal fiber, total protein, animal protein, and glycemic index. In addition, a previously identified association between nuts or seeds and diabetes (RR, 0.87; 95% CI, 0.81-0.94 per 4 servings/week that was based on 1 RCT and 5 cohort studies with 13 308 cases)^70^ was found to be no longer significant in more recent meta-analysis (RR, 0.89; 95% CI, 0.71-1.12 per 28 g/d, which was based on 7 cohort studies that included 15 470 cases).^63^

## Discussion

We identified probable or convincing quality of evidence for 15 dietary factors with protective associations^42,44,45,47,48,49,50,51,52,53,55,57,59,60,61,63,64,66^ and 10 dietary factors with harmful associations^5,46,47,54,56,58,62,65,67,68,71^ with specific CMD end points. Specifically, 20 dietary factors were associated with CVD, of which 12 were associated with diabetes or obesity and 7 were associated with both CVD and diabetes or obesity. Approximately three-quarters of the identified associations were for foods or beverages, and only one-quarter were for nutrients. The food, beverage, and nutrient associations were generally complementary and consistent. For example, fruits and vegetables as well as total dietary fiber, fruit fiber, and vegetable fiber were associated with CVD outcomes but not with diabetes. To our knowledge, this systematic review represents the most comprehensive current assessment of the quality of evidence for and the associations between dietary factors and CMD outcomes.

Several associations between diet and disease generated too few studies, reported insufficient information to enable the assessment of the Bradford-Hill criteria for causation, were null associations, had not been investigated in a dose-response meta-analysis, or met the criteria for probable or convincing evidence (eTable 2 in the Supplement). These dietary factors included important foods and beverages such as eggs, legumes, cheese, milk, and coffee, as well as nutrients such as dietary cholesterol, dietary calcium, and monounsaturated fatty acids. These findings highlight the lack of high-quality observational and experimental studies that are needed to identify the associations of diet with CMD and to evaluate the probable or convincing evidence of these associations.

Well-designed and executed RCTs are the most reliable means for drawing causality from associations, but not all associations between diet and disease can be ethically, feasibly, and appropriately examined in an RCT.^72,73,74^ Well-conducted observational studies can provide valid and reliable risk estimates for associations between diet and disease,^75^ but the quality of evidence for such associations should then be assessed for evidence for causal inference by using the 9 Bradford-Hill criteria for causation.^36^ The present assessment relied largely on meta-analyses of prospective observational studies except for the associations of saturated fatty acids^76^ with CVD and sodium with SBP,^5^ which were based on evidence from RCTs. However, this assessment also required confirmation of the Bradford-Hill criterion of experiment: supportive physiological evidence from RCTs in humans. Complementary evidence from observational studies and RCTs for intermediate risk factors or disease outcomes provides a scientific foundation for assessing the etiological factors in the association between diet and disease. For instance, RCTs in animals and humans showed that diets with higher glycemic index and glycemic load increased insulin resistance and abdominal or visceral obesity,^77,78,79^ a finding that was consistent with results from cohort studies of the associations of glycemic index and glycemic load with diabetes.^67^ Similarly, the association of polyunsaturated fatty acids with lower CHD risk was supported by cohort studies of blood biomarkers^80^ and RCTs of clinical events^81^ and blood lipids,^82^ and the association of polyunsaturated fatty acids with lower diabetes risk was supported by cohort studies of blood biomarkers^83^ and RCTs of glucose control and insulin resistance.^84^

Previous reviews have considered the associations between multiple dietary factors and CMD.^14,15,18,26,32,85^ However, few studies have formally assessed the quality of evidence, and only a few studies that have focused on the specific associations between diet and CMD, such as the association of glycemic index and glycemic load with risk of diabetes,^67,86^ have used the Bradford-Hill criteria for causation. Such studies found generally concordant findings as in the present work, identifying fruits, vegetables, whole grains, yogurt, and fiber as protective against the risk of some CMD outcomes, and unprocessed red meat, processed meat, and SSBs were regarded as harmful. Compared with an earlier review,^13^ the present study did not identify probable or convincing evidence of an association between nuts and diabetes and used meta-analyses that included new studies as well as potential differences in their methods (eg, multivariable adjustment for body mass index, which could be a mediator or confounder).^63,70^ Although nuts contain fiber, phenolics, and unsaturated fatty acids that would be expected to improve glucose control, the RCTs of nuts consumption have generally involved a small sample and shown mixed results.^87,88^

We believe this investigation builds on and expands the existing literature by providing a comprehensive summary of the current quality of evidence of the associations of dietary factors with CVD and diabetes using established criteria. The findings may inform dietary guidance, risk estimates and uncertainty to identify the disease burden for certain populations, policy setting to reduce the burden of diet-related CMD, and future research.

### Strengths and Limitations

This study has several strengths. Searches were broad and systematic, with inclusion and exclusion decisions and data extractions performed independently and in duplicate. We included meta-analyses of prospective cohort studies and RCTs, which are study designs with complementary strengths and limitations for assessing associations. Retrospective or cross-sectional studies, which increase risk of bias, were excluded. We assessed multiple dietary exposures, including major foods, beverages, and nutrients of clinical and public health interest. We used dose-response analyses, which incorporate all available data in a standardized fashion per serving size rather than comparing only extreme (eg, high vs low) and often heterogeneous categories of intake. The quality of evidence was formally evaluated using established Bradford-Hill criteria for causation.

This study also has potential limitations. Prospective cohort studies may be prone to residual confounding, which can bias results in different directions.^89^ However, we extracted multivariable-adjusted RR estimates, which generally included major confounders, and assessed the quality of evidence using the Bradford-Hill criteria. Both dietary intakes and clinical outcomes in large studies can be measured with error, which in prospective studies would generally attenuate the outcomes toward the null, leading to the underestimation of associations. Insufficient numbers of systematic reviews and meta-analyses were available to enable a rigorous investigation of the potential differences by subgroups (eg, sex, race and ethnicity, and world region); although large biological effect modification is generally rare, it cannot be ruled out. We did not assess the quality of individual studies given that many previous systematic reviews have done so using various criteria.^14,26,90^ We focused on broad dietary groupings, and there may be other relevant dietary factors that were not included, such as subtypes of fruits or vegetables. For the association of SSBs with body mass index change, we did not identify any published meta-analyses, and we selected the study included in a previous review,^13^ which pooled the findings from 3 large, prospective cohorts.^71^

## Conclusions

This systematic review summarized the quality of current evidence of the associations of specific dietary factors with CHD, stroke, and diabetes. These findings may inform dietary guidance, provide risk estimates and uncertainty to identify the disease burden for specific populations, help with policy setting to reduce the burden of diet-related CMD, and identify gaps in the literature to guide future research.

## Notes

Supplement. **eAppendix 1.** Criteria for Grading the Evidence of Associations of Specific Dietary Factors on Cardiometabolic Outcomes **eMethods.** Searches for Identifying Meta-analyses of the Associations of Specified Dietary Risk Factors on Cardiometabolic Diseases **eAppendix 2.** PubMed Search Terms **eTable 1.** Search Results, per Each Search Based on Types of Articles **eFigure 1.** Screening and Selection Process of Meta-analyses Evaluating Associations of Diet-Disease Relationships for Dietary Factors With Probable or Convincing Evidence on Cardiometabolic Diseases **eFigure 2.** Estimates of Etiologic Associations of Sodium and Systolic Blood Pressure **eTable 2.** Estimates of Associations of Dietary Factors and Risk of Cardiometabolic Disease (Original Units and RRs [CIs]) **eTable 3.** Reasons for Excluding Dietary Factor-CMD Relationships
eReferences
