# Supplementary material for: Evaluation of the Quality of Evidence of the Association of Foods and Nutrients With Cardiovascular Disease and Diabetes: A Systematic Review
Source: JAMA Netw Open. 2022 Feb 3;5(2):e2146705. doi: 10.1001/jamanetworkopen.2021.46705 (PMC8814912; doi:10.1001/jamanetworkopen.2021.46705)
Supplement: Supplement. — eAppendix 1. Criteria for Grading the Evidence of Associations of Specific Dietary Factors on Cardiometabolic Outcomes eMethods. Searches for Identifying Meta-analyses of the Associations of Specified Dietary Risk Factors on Cardiometabolic Diseases eAppendix 2. PubMed Search Terms eTable 1. Search Results, per Each Search Based on Types of Articles eFigure 1. Screening and Selection Process of Meta-analyses Evaluating Associations of Diet-Disease Relationships for Dietary Factors With Probable or Convincing Evidence on Cardiometabolic Diseases eFigure 2. Estimates of Etiologic Associations of Sodium and Systolic Blood Pressure eTable 2. Estimates of Associations of Dietary Factors and Risk of Cardiometabolic Disease (Original Units and RRs [CIs]) eTable 3. Reasons for Excluding Dietary Factor-CMD Relationships eReferences [file jamanetwopen-e2146705-s001.pdf]

Supplementary Online Content

Miller V, Micha R, Choi E, Karageorgou D, Webb P, Mozaffarian D. Evaluation of the quality of evidence of the association of foods and nutrients with cardiovascular disease and diabetes. *JAMA Netw Open*. 2022;5(2):e2146705. doi:10.1001/jamanetworkopen.2021.46705

eAppendix 1. Criteria for Grading the Evidence of Associations of Specific Dietary Factors on Cardiometabolic Outcomes
eMethod. Searches for Identifying Meta-analyses of the Associations of Specified Dietary Risk Factors on Cardiometabolic Diseases
eAppendix 2. PubMed Search Terms
eTable 1. Search Results, per Each Search Based on Types of Articles
eFigure 1. Screening and Selection Process of Meta-analyses Evaluating Associations of Diet-Disease Relationships for Dietary Factors With Probable or Convincing Evidence on Cardiometabolic Diseases
eFigure 2. Estimates of Etiologic Associations of Sodium and Systolic Blood Pressure
eTable 2. Estimates of Associations of Dietary Factors and Risk of Cardiometabolic Disease (Original Units and RRs [CIs])
eTable 3. Reasons for Excluding Dietary Factor-CMD Relationships
eReferences

This supplementary material has been provided by the authors to give readers additional information about their work.

© 2022 Miller V et al. *JAMA Network Open.*

eAppendix 1. Criteria for Grading the Evidence of Associations of Specific Dietary Factors on Cardiometabolic Outcomes

The following principles, focusing on meta-analyses of prospective cohort studies and/or randomized controlled trials, guided the scoring for each of 9 Bradford-Hill criteria:■ Consistent evidence from several well-designed studies with relatively few limitations;■ Consistent evidence from several studies but with some important limitations; ■ Emerging evidence from a few studies or conflicting results from several studies; - criterion not met. Definitions for each of the 9 criteria and adaptations to the general scoring system were as follows:

1. **Strength:** magnitude of association, including RRs for protective factors of >0.9 (■), 0.8-0.89 (■), or <0.8 (■); and for harmful factors, of <1.11 (■), 1.25 (■), and >1.25 (■). Since magnitude is directly dependent on both the selected serving size and frequency of consumption, we utilized serving sizes most similar to standard dietary guidelines and frequencies of consumption representing modest, standardized differences in intake (e.g., 1 serving/d of fruit) that are easily communicated and could be feasibly achieved by an intervention.
2. **Consistency**: association is repeatedly observed in different populations and circumstances, including ≥80% of included study-specific estimates being in the expected direction (■); ≥60 - <80% (■); ≥40 - <60% (■); and <40% (not meeting criteria). (Though some other grading frameworks use statistical heterogeneity, this is not optimal to assess consistency as characterized by Bradford-Hill. Statistical measures of heterogeneity are influenced by both magnitudes of differences and also the numbers of studies and precision of each estimate. Thus, diet-disease relationships with few studies could have lack of consistency but fail to achieve statistical heterogeneity due to low power; while diet-disease relationships having many studies with high precision could exhibit statistical heterogeneity yet still be consistent in terms of their overall inference for the effect of the dietary factor on disease.)
3. **Temporality:** exposure precedes outcome. Because all evidence was based on longitudinal studies, this was a necessary criterion (■); when relatively few overall studies were available (<5), we graded this criterion conservatively as ■.
4. **Coherence:** interpretation of association does not conflict with known natural history and biology of the disease, for example based on pathways of disease occurrence and laboratory findings on the dietary factor.
5. **Specificity:** exposure linked to a specific outcome. Because many nutritional factors can plausibly have diverse effects and influence multiple outcomes, scoring was based on three

© 2022 Miller V et al. *JAMA Network Open.*

principles: 1) dietary factor influences a mechanism/pathways known to cause the outcome; 2) dietary factor not associated with multiple other, unrelated non-communicable diseases (e.g., multiple cancers, chronic obstructive pulmonary disease (COPD)); 3) dietary association has additional specificity within the set of cardiometabolic outcomes (coronary heart disease (CHD), stroke, diabetes mellitus).

6. **Analogy:** based on the effects of similar factors on the disease outcome:

Fruit, vegetables, nuts/seeds: based on analogies with other minimally processed, higher fiber, phytochemical rich foods.

Potatoes: based on analogies to other higher glycemic load carbohydrates in relation to both diabetes mellitus and weight grain (don't want to refer to potatoes as poor-quality carbohydrates).

Whole grains: based on analogies with other less-processed foods, dietary fiber, and glycemic load.

Unprocessed red meat with CVD: based on analogies to dietary heme iron to myocardial infarction and fatal coronary heart disease, L-carnitine, and trimethylamine-N-oxide.

Unprocessed red meat and processed meat with diabetes: based on analogies to processed meats (or unprocessed red meats), blood ferritin levels, and hemochromatosis.

Processed meat with CVD: based on analogies to sodium.

Fish/seafood: based on analogies to omega-3 fatty acids.

Yogurt: based on analogies to probiotics in relation to weight gain.

Chocolate and tea: based on analogies to other polyphenol rich foods.

Milk: based on analogies to other dairy products.

Sugar sweetened beverages, glycemic index, and glycemic load with CVD: based on analogies to other poor-quality carbohydrates in relation to both CVD and weight gain.

Sugar sweetened beverages, glycemic index, and glycemic load with diabetes: based on analogies to other poor-quality carbohydrates in relation to both diabetes mellitus and weight gain.

Polyunsaturated fat: based on analogies to vegetable oils in relation to CHD and cardiovascular risk factors.

Trans-fat: based on analogies to other dietary fats.

Total protein and animal protein: based on analogies to protein rich foods.

Sodium and potassium with CVD: based on analogies to other lifestyle-related and nonlifestyle-related blood pressure interventions and to foods high in sodium (eg, processed meats).

Sodium with blood pressure: based on analogies to potassium.

7. **Plausibility:** association supported by one or more credible biological mechanisms.

8. **Biological gradient:** exposure and outcome are related by a monotonic dose-response curve.

© 2022 Miller V et al. *JAMA Network Open.*

9. **Experiment:** association is also supported by evidence from randomized controlled trials on intermediate risk factors (or, less commonly, disease outcomes) plus supportive laboratory studies.

Fruit and vegetables: because while strong and consistent evidence from trials of dietary patterns rich in fruits and vegetables, few trials separately evaluated only fruits or vegetables.

Potatoes: based on findings from trials on weight gain and insulin resistance.

Whole grains with diabetes: based on overall effects of carbohydrate quality, including studies of dietary fiber and glycemic load; much less evidence for benefits of whole grains independent of dietary fiber and glycemic load.

Fish with CHD in diabetes patients: based on trials on effects of fish oil supplements on cardiovascular disease in diabetic patients.

Yogurt: based on findings for yogurt and weight gain (animal studies, human cohorts) and for probiotics and weight gain (animal and human experiments).

Chocolate with CVD: based on trials on chocolate and blood pressure.

Tea: based on findings from trials on tea and blood pressure, LDL cholesterol or glycemic response.

Following grading of Bradford-Hill criteria, the characterization of overall sufficient probable or convincing evidence for each diet-disease relationship was based on independent review by two investigators (VM, DM) of the overall findings across the Bradford-Hill criteria,[1] with additional guidance from other definitions for probable or convincing evidence of causality from the WHO and WCRF/AICR;[2-4] any differences were resolved by consensus.

© 2022 Miller V et al. *JAMA Network Open.*

eMethod. Searches for Identifying Meta-analyses of the Associations of Specified Dietary Risk Factors on Cardiometabolic Diseases

For each identified diet-disease relationship, we performed multiple systematic searches of PubMed to identify meta-analyses of randomized controlled trials or prospective cohort studies evaluating these specific dietary factors and total cardiovascular disease, coronary heart disease, stroke including subtypes (ischemic, hemorrhagic), or diabetes. For sodium, and sugar-sweetened beverages and non-nutritive sweetened beverages, we also reviewed effects on blood pressure and obesity, respectively, based on randomized trials demonstrating primary effects on these risk pathways. We did not search for individual papers/studies across multiple dietary risk factors and outcomes, rather we only included published, peer-reviewed meta-analyses. Based on our and other recent reviews[5,6], we did not include multiple other factors for which the initial appraisal identified one or more key limitations that would limit meeting the criteria for quality of evidence. Due to the multiple dietary factors evaluated, a formal listing of the specific reasons for the exclusion of each was not recorded. For each included meta-analysis, we extracted the multivariable-adjusted effect estimate and corresponding 95% confidence intervals (CIs), which generally included major confounders, and did not recalculate the effect estimate by including only adjusted estimates or individual studies adjusting for certain confounders.

© 2022 Miller V et al. *JAMA Network Open.*

eAppendix 2. PubMed Search Terms

Limits:

Age: Any

Setting: Any country

Year Range: Any

Language: English

Species: Human

Type of Article: Meta-Analysis [ptyp] OR Meta-Analysis [tiab] OR "Systematic Review"[tiab]

Note: if a search term exists as Mesh, then use both ([mesh] OR [tiab]); if only free-text searching (i.e., no Mesh) then use [tiab]

Date: May/1/2015 through February/26/2021 for all diet factors

Dietary Factors:

Foods

Fruits

Fruit juices

Vegetables

Potatoes

Beans/legumes

Nuts/seeds

Whole grains

Refined grains

Milk

Yogurt

Cheese

Unprocessed red meats

Processed meats

Fish/seafood

Total fish/seafood

Lean fish

Fatty fish

Eggs

Sugar-sweetened beverages

Non-nutritive sweetened beverages

Coffee

Tea

© 2022 Miller V et al. *JAMA Network Open.*

Cocoa (chocolate)

Nutrients

Dietary fatty acids

Polyunsaturated fats (PUFA)

Saturated fats (SFA)

Monounsaturated fats (MUFA)

Seafood omega-3 fats

Plant omega-3 fats

Trans fats

Dietary protein

Total protein

Animal protein

Plant protein

Dietary cholesterol

Dietary fiber

Total fiber

Cereal fiber

Fruit fiber

Vegetable fiber

Legume fiber

Glycemic index (GI)

Glycemic load (GL)

Dietary sodium

Dietary potassium

Dietary calcium

Energy

Outcomes:

Total or non-fatal cardiovascular disease (CVD)

Total or non-fatal coronary heart disease (CHD), coronary artery disease (CAD) or ischemic heart disease (IHD)

Total or non-fatal myocardial infarction (MI)

Total or non-fatal stroke

Total or non-fatal ischemic stroke

Total or non-fatal hemorrhagic stroke

Type II diabetes mellitus

© 2022 Miller V et al. *JAMA Network Open.*

Change in body mass index (BMI) [sugar-sweetened beverages and non-nutritive sweetened beverages only]

Systolic blood pressure (SBP) [sodium only]

Diastolic blood pressure (DBP) [sodium only]

© 2022 Miller V et al. *JAMA Network Open.*

Inclusion Criteria

1. **Study Design:** Systematic review/meta-analysis of prospective cohort studies (including nested case-control design) or randomized controlled trials.
2. **Population:** General adult human population 18 years or older; adult populations with prediabetes, insulin resistance, metabolic syndrome, hypertension, overweight or obesity; adults with type 2 diabetes (CVD outcomes only).
3. **Exposure:** Intake of specified dietary factors, either continuous or in >2 categories of intake to allow for adequate categorization of intake.
4. **Outcome:** Incident (fatal or non-fatal) of specified cardiometabolic health outcomes.
5. **Effect Estimate:** Multi-variate adjusted effect estimate (OR, RR, HR) and variance.
6. **Setting:** No restrictions on type of setting applied.
7. **Language:** English.
8. **Publication type:** Full text, published, peer reviewed.

Exclusion Criteria

1. **Study Design:** Systematic review/meta-analysis of retrospective case-control studies; pooling studies/genome-wide association studies (GWAS).
2. **Population:** Younger than 18 years old, pregnant women, children, and specified diseased (e.g., end-stage or rare diseases) or special (e.g., vegetarians vs. non-vegetarians) populations.
3. **Exposure:** mixed healthy diet was reported, where the individual dietary factor could not be separated; dietary supplementation or biomarker studies/trials.
4. **Outcome:** Studies of prevalence of outcome, studies of intermediate risk factors to the outcomes of interest (e.g., lipids, hypertension).
5. **Effect Estimate:** unadjusted (crude) risk estimates only.
6. **Number of primary studies:** <3 individual studies in the dose-response meta-analysis.
7. **Duplicate Publications:** When duplicate publications were identified, the report on the largest number of cases for each endpoint of interest and/or most recent search date was included.

© 2022 Miller V et al. *JAMA Network Open.*

Fruit search – 266 hits
(“fruit” [MeSH] OR “fruit” [tiab] OR “fruits” [tiab]) **AND** (“Cardiovascular Diseases” [MeSH] OR “Cardiovascular Disease” [tiab] OR “Cardiovascular Diseases” [tiab] OR “Heart Diseases” [MeSH] OR “Heart Diseases” [tiab] OR "myocardial infarction"[tiab] OR "myocardial infarctions"[tiab] OR "heart attack"[tiab] OR "heart attacks"[tiab] OR "sudden death"[tiab] OR "sudden deaths"[tiab] OR stroke[tiab] OR strokes[tiab] OR "cerebrovascular accident"[tiab] OR "cerebrovascular accidents"[tiab] OR “Diabetes Mellitus” [MeSH] OR “Diabetes Mellitus” [tiab] OR “Diabetes” [tiab] OR “Diabetes Mellitus, Type 2” [MeSH]) **AND (**“Meta-Analysis” [ptyp] OR “Meta-Analysis” [tiab] OR “Meta-analyses” [tiab] OR "Systematic Review" [tiab] OR "Systematic literature Review"[tiab] OR "Comprehensive Review"[tiab] OR "Comprehensive literature Review” [tiab]**) AND (**"2015/05/01” [PDat]: "2021/02/26” [PDat]**)**

Fruit juice search – 40 hits
(“fruit juice” [tiab] OR “fruit juices” [tiab] OR “juice”[tiab]) **AND** (“Cardiovascular Diseases” [MeSH] OR “Cardiovascular Disease” [tiab] OR “Cardiovascular Diseases” [tiab] OR “Heart Diseases” [MeSH] OR “Heart Diseases” [tiab] OR "myocardial infarction"[tiab] OR "myocardial infarctions"[tiab] OR "heart attack"[tiab] OR "heart attacks"[tiab] OR "sudden death"[tiab] OR "sudden deaths"[tiab] OR stroke[tiab] OR strokes[tiab] OR "cerebrovascular accident"[tiab] OR "cerebrovascular accidents"[tiab] OR “Diabetes Mellitus” [MeSH] OR “Diabetes Mellitus” [tiab] OR “Diabetes” [tiab] OR “Diabetes Mellitus, Type 2” [MeSH]) **AND (**“Meta-Analysis” [ptyp] OR “Meta-Analysis” [tiab] OR “Meta-analyses” [tiab] OR "Systematic Review" [tiab] OR "Systematic literature Review"[tiab] OR "Comprehensive Review"[tiab] OR "Comprehensive literature Review” [tiab]**) AND (**"2015/05/01” [PDat]: "2021/02/26” [PDat]**)**

Vegetable search – 199 hits
(“Vegetables” [MeSH] OR “Vegetables” [tiab] OR “Vegetable” [tiab]) **AND** (“Cardiovascular Diseases” [MeSH] OR “Cardiovascular Disease” [tiab] OR “Cardiovascular Diseases” [tiab] OR “Heart Diseases” [MeSH] OR “Heart Diseases” [tiab] OR "myocardial infarction"[tiab] OR "myocardial infarctions"[tiab] OR "heart attack"[tiab] OR "heart attacks"[tiab] OR "sudden death"[tiab] OR "sudden deaths"[tiab] OR stroke[tiab] OR strokes[tiab] OR "cerebrovascular accident"[tiab] OR "cerebrovascular accidents"[tiab] OR “Diabetes Mellitus” [MeSH] OR “Diabetes Mellitus” [tiab] OR “Diabetes” [tiab] OR “Diabetes Mellitus, Type 2” [MeSH]) **AND (**“Meta-Analysis” [ptyp] OR “Meta-Analysis” [tiab] OR “Meta-analyses” [tiab] OR "Systematic Review" [tiab] OR "Systematic literature Review"[tiab] OR "Comprehensive Review"[tiab] OR "Comprehensive literature Review” [tiab]**) AND (**"2015/05/01” [PDat]: "2021/02/26” [PDat]**)**

© 2022 Miller V et al. *JAMA Network Open.*

Potato search – 13 hits

(“potato” [Mesh] OR “potato” [tiab]) **AND** (“Cardiovascular Diseases” [MeSH] OR “Cardiovascular Disease” [tiab] OR “Cardiovascular Diseases” [tiab] OR “Heart Diseases” [MeSH] OR “Heart Diseases” [tiab] OR "myocardial infarction"[tiab] OR "myocardial infarctions"[tiab] OR "heart attack"[tiab] OR "heart attacks"[tiab] OR "sudden death"[tiab] OR "sudden deaths"[tiab] OR stroke[tiab] OR strokes[tiab] OR "cerebrovascular accident"[tiab] OR "cerebrovascular accidents"[tiab] OR “Diabetes Mellitus” [MeSH] OR “Diabetes Mellitus” [tiab] OR “Diabetes” [tiab] OR “Diabetes Mellitus, Type 2” [MeSH]) **AND (**“Meta-Analysis” [ptyp] OR “Meta-Analysis” [tiab] OR “Meta-analyses” [tiab] OR "Systematic Review" [tiab] OR "Systematic literature Review"[tiab] OR "Comprehensive Review"[tiab] OR "Comprehensive literature Review” [tiab]**) AND (**"2015/05/01” [PDat]: "2021/02/26” [PDat]**)**

Beans/legumes search – 59 hits

(“Bean” [tiab] OR “Beans” [tiab] OR “legumes” [tiab] OR “legumes” [tiab]) **AND** (“Cardiovascular Diseases” [MeSH] OR “Cardiovascular Disease” [tiab] OR “Cardiovascular Diseases” [tiab] OR “Heart Diseases” [MeSH] OR “Heart Diseases” [tiab] OR "myocardial infarction"[tiab] OR "myocardial infarctions"[tiab] OR "heart attack"[tiab] OR "heart attacks"[tiab] OR "sudden death"[tiab] OR "sudden deaths"[tiab] OR stroke[tiab] OR strokes[tiab] OR "cerebrovascular accident"[tiab] OR "cerebrovascular accidents"[tiab] OR “Diabetes Mellitus” [MeSH] OR “Diabetes Mellitus” [tiab] OR “Diabetes” [tiab] OR “Diabetes Mellitus, Type 2” [MeSH]) **AND (**“Meta-Analysis” [ptyp] OR “Meta-Analysis” [tiab] OR “Meta-analyses” [tiab] OR "Systematic Review" [tiab] OR "Systematic literature Review"[tiab] OR "Comprehensive Review"[tiab] OR "Comprehensive literature Review” [tiab]**) AND (**"2015/05/01” [PDat]: "2021/02/26” [PDat]**)**

Nuts/seeds search – 178 hits

(“Nuts” [MeSH] OR “Nuts” [tiab] OR “Nut” [tiab] OR “Seeds” [MeSH] OR “Seeds” [tiab] OR “Seed” [tiab]) **AND** (“Cardiovascular Diseases” [MeSH] OR “Cardiovascular Disease” [tiab] OR “Cardiovascular Diseases” [tiab] OR “Heart Diseases” [MeSH] OR “Heart Diseases” [tiab] OR "myocardial infarction"[tiab] OR "myocardial infarctions"[tiab] OR "heart attack"[tiab] OR "heart attacks"[tiab] OR "sudden death"[tiab] OR "sudden deaths"[tiab] OR stroke[tiab] OR strokes[tiab] OR "cerebrovascular accident"[tiab] OR "cerebrovascular accidents"[tiab] OR “Diabetes Mellitus” [MeSH] OR “Diabetes Mellitus” [tiab] OR “Diabetes” [tiab] OR “Diabetes Mellitus, Type 2” [MeSH]) **AND (**“Meta-Analysis” [ptyp] OR “Meta-Analysis” [tiab] OR “Meta-

© 2022 Miller V et al. *JAMA Network Open.*

analyses” [tiab] OR "Systematic Review" [tiab] OR "Systematic literature Review"[tiab] OR "Comprehensive Review"[tiab] OR "Comprehensive literature Review” [tiab]**) AND (**"2015/05/01” [PDat]: "2021/02/26” [PDat]**)**

Whole grains search – 71 hits
(“Whole grains” [tiab] OR “Whole grain” [tiab]) **AND** (“Cardiovascular Diseases” [MeSH] OR “Cardiovascular Disease” [tiab] OR “Cardiovascular Diseases” [tiab] OR “Heart Diseases” [MeSH] OR “Heart Diseases” [tiab] OR "myocardial infarction"[tiab] OR "myocardial infarctions"[tiab] OR "heart attack"[tiab] OR "heart attacks"[tiab] OR "sudden death"[tiab] OR "sudden deaths"[tiab] OR stroke[tiab] OR strokes[tiab] OR "cerebrovascular accident"[tiab] OR "cerebrovascular accidents"[tiab] OR “Diabetes Mellitus” [MeSH] OR “Diabetes Mellitus” [tiab] OR “Diabetes” [tiab] OR “Diabetes Mellitus, Type 2” [MeSH]) **AND (**“Meta-Analysis” [ptyp] OR “Meta-Analysis” [tiab] OR “Meta-analyses” [tiab] OR "Systematic Review" [tiab] OR "Systematic literature Review"[tiab] OR "Comprehensive Review"[tiab] OR "Comprehensive literature Review” [tiab]**) AND (**"2015/05/01” [PDat]: "2021/02/26” [PDat]**)**

Refined grains search – 23 hits
(“Refined grains” [tiab] OR “Refined grain” [tiab]) **AND** (“Cardiovascular Diseases” [MeSH] OR “Cardiovascular Disease” [tiab] OR “Cardiovascular Diseases” [tiab] OR “Heart Diseases” [MeSH] OR “Heart Diseases” [tiab] OR "myocardial infarction"[tiab] OR "myocardial infarctions"[tiab] OR "heart attack"[tiab] OR "heart attacks"[tiab] OR "sudden death"[tiab] OR "sudden deaths"[tiab] OR stroke[tiab] OR strokes[tiab] OR "cerebrovascular accident"[tiab] OR "cerebrovascular accidents"[tiab] OR “Diabetes Mellitus” [MeSH] OR “Diabetes Mellitus” [tiab] OR “Diabetes” [tiab] OR “Diabetes Mellitus, Type 2” [MeSH]) **AND (**“Meta-Analysis” [ptyp] OR “Meta-Analysis” [tiab] OR “Meta-analyses” [tiab] OR "Systematic Review" [tiab] OR "Systematic literature Review"[tiab] OR "Comprehensive Review"[tiab] OR "Comprehensive literature Review” [tiab]**) AND (**"2015/05/01” [PDat]: "2021/02/26” [PDat]**)**

Dairy search – 181 hits
(“Dairy Products” [MeSH] OR “dairy” [tiab] OR “cheese” [MeSH] OR “cheese” [tiab] OR “yogurt” [MeSH] OR “yogurt” [tiab] OR “yoghurt” [tiab] OR “milk” [MeSH] OR “milk”[tiab]) **AND** (“Cardiovascular Diseases” [MeSH] OR “Cardiovascular Disease” [tiab] OR “Cardiovascular Diseases” [tiab] OR “Heart Diseases” [MeSH] OR “Heart Diseases” [tiab] OR "myocardial infarction"[tiab] OR "myocardial infarctions"[tiab] OR "heart attack"[tiab] OR "heart attacks"[tiab] OR "sudden death"[tiab] OR "sudden deaths"[tiab] OR stroke[tiab] OR

strokes[tiab] OR "cerebrovascular accident"[tiab] OR "cerebrovascular accidents"[tiab] OR "Diabetes Mellitus" [MeSH] OR "Diabetes Mellitus" [tiab] OR "Diabetes" [tiab] OR "Diabetes Mellitus, Type 2" [MeSH]) **AND (**"Meta-Analysis" [ptyp] OR "Meta-Analysis" [tiab] OR "Meta-analyses" [tiab] OR "Systematic Review" [tiab] OR "Systematic literature Review"[tiab] OR "Comprehensive Review"[tiab] OR "Comprehensive literature Review" [tiab]**) AND (**"2015/05/01" [PDat]: "2021/02/26" [PDat]**)**

Unprocessed red meat and processed meat search – 112 hits
("meat" [Mesh] OR "meat" [tiab]) **AND** ("Cardiovascular Diseases" [MeSH] OR "Cardiovascular Disease" [tiab] OR "Cardiovascular Diseases" [tiab] OR "Heart Diseases" [MeSH] OR "Heart Diseases" [tiab] OR "myocardial infarction"[tiab] OR "myocardial infarctions"[tiab] OR "heart attack"[tiab] OR "heart attacks"[tiab] OR "sudden death"[tiab] OR "sudden deaths"[tiab] OR stroke[tiab] OR strokes[tiab] OR "cerebrovascular accident"[tiab] OR "cerebrovascular accidents"[tiab] OR "Diabetes Mellitus" [MeSH] OR "Diabetes Mellitus" [tiab] OR "Diabetes" [tiab] OR "Diabetes Mellitus, Type 2" [MeSH]) **AND (**"Meta-Analysis" [ptyp] OR "Meta-Analysis" [tiab] OR "Meta-analyses" [tiab] OR "Systematic Review" [tiab] OR "Systematic literature Review"[tiab] OR "Comprehensive Review"[tiab] OR "Comprehensive literature Review" [tiab]**) AND (**"2015/05/01" [PDat]: "2021/02/26" [PDat]**)**

Fish search – 129 hits
("fishes" [MeSH] OR "fishes" [tiab] OR "fish" [tiab]) **AND** ("Cardiovascular Diseases" [MeSH] OR "Cardiovascular Disease" [tiab] OR "Cardiovascular Diseases" [tiab] OR "Heart Diseases" [MeSH] OR "Heart Diseases" [tiab] OR "myocardial infarction"[tiab] OR "myocardial infarctions"[tiab] OR "heart attack"[tiab] OR "heart attacks"[tiab] OR "sudden death"[tiab] OR "sudden deaths"[tiab] OR stroke[tiab] OR strokes[tiab] OR "cerebrovascular accident"[tiab] OR "cerebrovascular accidents"[tiab] OR "Diabetes Mellitus" [MeSH] OR "Diabetes Mellitus" [tiab] OR "Diabetes" [tiab] OR "Diabetes Mellitus, Type 2" [MeSH]) **AND (**"Meta-Analysis" [ptyp] OR "Meta-Analysis" [tiab] OR "Meta-analyses" [tiab] OR "Systematic Review" [tiab] OR "Systematic literature Review"[tiab] OR "Comprehensive Review"[tiab] OR "Comprehensive literature Review" [tiab]**) AND (**"2015/05/01" [PDat]: "2021/02/26" [PDat]**)**

Eggs search – 40 hits
("eggs" [MeSH] OR "eggs" [tiab]) **AND** ("Cardiovascular Diseases" [MeSH] OR "Cardiovascular Disease" [tiab] OR "Cardiovascular Diseases" [tiab] OR "Heart Diseases"

© 2022 Miller V et al. *JAMA Network Open.*

[MeSH] OR “Heart Diseases” [tiab] OR "myocardial infarction"[tiab] OR "myocardial infarctions"[tiab] OR "heart attack"[tiab] OR "heart attacks"[tiab] OR "sudden death"[tiab] OR "sudden deaths"[tiab] OR stroke[tiab] OR strokes[tiab] OR "cerebrovascular accident"[tiab] OR "cerebrovascular accidents"[tiab] OR “Diabetes Mellitus” [MeSH] OR “Diabetes Mellitus” [tiab] OR “Diabetes” [tiab] OR “Diabetes Mellitus, Type 2” [MeSH]) **AND (**“Meta-Analysis” [ptyp] OR “Meta-Analysis” [tiab] OR “Meta-analyses” [tiab] OR "Systematic Review" [tiab] OR "Systematic literature Review"[tiab] OR "Comprehensive Review"[tiab] OR "Comprehensive literature Review” [tiab]**) AND (**"2015/05/01” [PDat]: "2021/02/26” [PDat]**)**

Sugar-sweetened beverages search- 378 hits

(“Sugar-sweetened beverages” [tiab] OR “Sugar-sweetened beverage” [tiab] OR “Sugar sweetened beverages” [tiab] OR “Sugar sweetened beverage” [tiab] OR “beverages” [MeSH] OR “beverages” [tiab] OR “beverage” [tiab]) **AND** (“Cardiovascular Diseases” [MeSH] OR “Cardiovascular Disease” [tiab] OR “Cardiovascular Diseases” [tiab] OR “Heart Diseases” [MeSH] OR “Heart Diseases” [tiab] OR "myocardial infarction"[tiab] OR "myocardial infarctions"[tiab] OR "heart attack"[tiab] OR "heart attacks"[tiab] OR "sudden death"[tiab] OR "sudden deaths"[tiab] OR stroke[tiab] OR strokes[tiab] OR "cerebrovascular accident"[tiab] OR "cerebrovascular accidents"[tiab] OR “Diabetes Mellitus” [MeSH] OR “Diabetes Mellitus” [tiab] OR “Diabetes” [tiab] OR “Diabetes Mellitus, Type 2” [MeSH] OR “obesity” [MeSH] OR “obesity” [tiab] OR “overweight” [MeSH] OR “overweight” [tiab] OR “body mass index” [MeSH] OR “body mass index” [tiab]) **AND (**“Meta-Analysis” [ptyp] OR “Meta-Analysis” [tiab] OR “Meta-analyses” [tiab] OR "Systematic Review" [tiab] OR "Systematic literature Review"[tiab] OR "Comprehensive Review"[tiab] OR "Comprehensive literature Review” [tiab]**) AND (**"2015/05/01” [PDat]: "2021/02/26” [PDat]**)**

Non-nutritive sweetened beverages search- 10 hits

(“Non-nutritive sweetened beverages” [tiab] OR “Non nutritive sweetened beverage” [tiab] OR “non nutritive sweetened beverage” [tiab] OR “non nutritive sweetener” [MeSH] OR “artificial sweetener” [tiab] OR “artificially sweetened beverage” [tiab] OR “artificially sweetened” [tiab] OR “low calorie beverage” [tiab]) OR “sugar free beverage” [tiab]) OR “reduced sugar beverage” [tiab]) **AND** (“Cardiovascular Diseases” [MeSH] OR “Cardiovascular Disease” [tiab] OR “Cardiovascular Diseases” [tiab] OR “Heart Diseases” [MeSH] OR “Heart Diseases” [tiab] OR "myocardial infarction"[tiab] OR "myocardial infarctions"[tiab] OR "heart attack"[tiab] OR "heart attacks"[tiab] OR "sudden death"[tiab] OR "sudden deaths"[tiab] OR stroke[tiab] OR

© 2022 Miller V et al. *JAMA Network Open.*

strokes[tiab] OR "cerebrovascular accident"[tiab] OR "cerebrovascular accidents"[tiab] OR "Diabetes Mellitus" [MeSH] OR "Diabetes Mellitus" [tiab] OR "Diabetes" [tiab] OR "Diabetes Mellitus, Type 2" [MeSH] OR "obesity" [MeSH] OR "obesity" [tiab] OR "overweight" [MeSH] OR "overweight" [tiab] OR "body mass index" [MeSH] OR "body mass index" [tiab]) **AND** **(**"Meta-Analysis" [ptyp] OR "Meta-Analysis" [tiab] OR "Meta-analyses" [tiab] OR "Systematic Review" [tiab] OR "Systematic literature Review"[tiab] OR "Comprehensive Review"[tiab] OR "Comprehensive literature Review" [tiab]**)** **AND** **(**"2015/05/01" [PDat]: "2021/02/26" [PDat]**)**

Coffee and tea search – 116 hits
("coffee" [MeSH] OR "coffee" [tiab] OR "tea" [MeSH] OR "tea" [tiab]) **AND** ("Cardiovascular Diseases" [MeSH] OR "Cardiovascular Disease" [tiab] OR "Cardiovascular Diseases" [tiab] OR "Heart Diseases" [MeSH] OR "Heart Diseases" [tiab] OR "myocardial infarction"[tiab] OR "myocardial infarctions"[tiab] OR "heart attack"[tiab] OR "heart attacks"[tiab] OR "sudden death"[tiab] OR "sudden deaths"[tiab] OR stroke[tiab] OR strokes[tiab] OR "cerebrovascular accident"[tiab] OR "cerebrovascular accidents"[tiab] OR "Diabetes Mellitus" [MeSH] OR "Diabetes Mellitus" [tiab] OR "Diabetes" [tiab] OR "Diabetes Mellitus, Type 2" [MeSH]) **AND** **(**"Meta-Analysis" [ptyp] OR "Meta-Analysis" [tiab] OR "Meta-analyses" [tiab] OR "Systematic Review" [tiab] OR "Systematic literature Review"[tiab] OR "Comprehensive Review"[tiab] OR "Comprehensive literature Review" [tiab]**)** **AND** **(**"2015/05/01" [PDat]: "2021/02/26" [PDat]**)**

Cocoa search – 26 hits
("Cacao"[MeSH] OR "Cacao"[tiab] OR "cocoa" [tiab] OR ("dark"[tiab] AND "chocolate"[tiab])) **AND** ("Cardiovascular Diseases" [MeSH] OR "Cardiovascular Disease" [tiab] OR "Cardiovascular Diseases" [tiab] OR "Heart Diseases" [MeSH] OR "Heart Diseases" [tiab] OR "myocardial infarction"[tiab] OR "myocardial infarctions"[tiab] OR "heart attack"[tiab] OR "heart attacks"[tiab] OR "sudden death"[tiab] OR "sudden deaths"[tiab] OR stroke[tiab] OR strokes[tiab] OR "cerebrovascular accident"[tiab] OR "cerebrovascular accidents"[tiab] OR "Diabetes Mellitus" [MeSH] OR "Diabetes Mellitus" [tiab] OR "Diabetes" [tiab] OR "Diabetes Mellitus, Type 2" [MeSH]) **AND** **(**"Meta-Analysis" [ptyp] OR "Meta-Analysis" [tiab] OR "Meta-analyses" [tiab] OR "Systematic Review" [tiab] OR "Systematic literature Review"[tiab] OR "Comprehensive Review"[tiab] OR "Comprehensive literature Review" [tiab]**)** **AND** **(**"2015/05/01" [PDat]: "2021/02/26" [PDat]**)**

© 2022 Miller V et al. *JAMA Network Open.*

Dietary protein search – 114 hits

((“Protein” [MeSH] AND "diet"[MeSH)) OR (“dietary” [tiab] AND “Protein” [tiab])) **AND** (“Cardiovascular Diseases” [MeSH] OR “Cardiovascular Disease” [tiab] OR “Cardiovascular Diseases” [tiab] OR “Heart Diseases” [MeSH] OR “Heart Diseases” [tiab] OR "myocardial infarction"[tiab] OR "myocardial infarctions"[tiab] OR "heart attack"[tiab] OR "heart attacks"[tiab] OR "sudden death"[tiab] OR "sudden deaths"[tiab] OR stroke[tiab] OR strokes[tiab] OR "cerebrovascular accident"[tiab] OR "cerebrovascular accidents"[tiab] OR “Diabetes Mellitus” [MeSH] OR “Diabetes Mellitus” [tiab] OR “Diabetes” [tiab] OR “Diabetes Mellitus, Type 2” [MeSH]) **AND (**“Meta-Analysis” [ptyp] OR “Meta-Analysis” [tiab] OR “Meta-analyses” [tiab] OR "Systematic Review" [tiab] OR "Systematic literature Review"[tiab] OR "Comprehensive Review"[tiab] OR "Comprehensive literature Review” [tiab]**) AND (**"2015/05/01” [PDat]: "2021/02/26” [PDat]**)**

Dietary fatty acids search – 850 hits

("Fatty Acids, Omega-6"[MeSH] OR "Fatty Acids, Omega-3"[MeSH] OR "Fatty Acids, Unsaturated"[MeSH] OR "Fatty Acids, Monounsaturated"[MeSH] OR "Trans Fatty Acids"[MeSH] OR "monounsaturated"[tiab] OR "mono-unsaturated"[tiab] OR "MUFA"[tiab] OR "unsaturated"[tiab] OR "polyunsaturated"[tiab] OR "PUFA"[tiab] OR "saturated"[tiab] OR "SFA"[tiab] OR "trans-unsaturated"[tiab] OR "trans-fatty"[tiab] OR "trans fatty"[tiab] OR "trans unsaturated"[tiab] OR "trans fat"[tiab] OR "TFA"[tiab] OR "omega-6"[tiab] OR "omega-3"[tiab] OR "n-6"[tiab] OR "n-3"[tiab] OR “alpha-linolenic”[tiab] OR "oleic"[tiab] OR "linoleic"[tiab]) **AND** (“Cardiovascular Diseases” [MeSH] OR “Cardiovascular Disease” [tiab] OR “Cardiovascular Diseases” [tiab] OR “Heart Diseases” [MeSH] OR “Heart Diseases” [tiab] OR "myocardial infarction"[tiab] OR "myocardial infarctions"[tiab] OR "heart attack"[tiab] OR "heart attacks"[tiab] OR "sudden death"[tiab] OR "sudden deaths"[tiab] OR stroke[tiab] OR strokes[tiab] OR "cerebrovascular accident"[tiab] OR "cerebrovascular accidents"[tiab] OR “Diabetes Mellitus” [MeSH] OR “Diabetes Mellitus” [tiab] OR “Diabetes” [tiab] OR “Diabetes Mellitus, Type 2” [MeSH]) **AND (**“Meta-Analysis” [ptyp] OR “Meta-Analysis” [tiab] OR “Meta-analyses” [tiab] OR "Systematic Review" [tiab] OR "Systematic literature Review"[tiab] OR "Comprehensive Review"[tiab] OR "Comprehensive literature Review” [tiab]**) AND (**"2015/05/01” [PDat]: "2021/02/26” [PDat]**)**

Dietary cholesterol search – 16 hits

(“Cholesterol, Dietary” [MeSH] OR “Dietary cholesterol” [tiab]) **AND** (“Cardiovascular Diseases” [MeSH] OR “Cardiovascular Disease” [tiab] OR “Cardiovascular Diseases” [tiab] OR

“Heart Diseases” [MeSH] OR “Heart Diseases” [tiab] OR "myocardial infarction"[tiab] OR "myocardial infarctions"[tiab] OR "heart attack"[tiab] OR "heart attacks"[tiab] OR "sudden death"[tiab] OR "sudden deaths"[tiab] OR stroke[tiab] OR strokes[tiab] OR "cerebrovascular accident"[tiab] OR "cerebrovascular accidents"[tiab] OR “Diabetes Mellitus” [MeSH] OR “Diabetes Mellitus” [tiab] OR “Diabetes” [tiab] OR “Diabetes Mellitus, Type 2” [MeSH]) **AND (**“Meta-Analysis” [ptyp] OR “Meta-Analysis” [tiab] OR “Meta-analyses” [tiab] OR "Systematic Review" [tiab] OR "Systematic literature Review"[tiab] OR "Comprehensive Review"[tiab] OR "Comprehensive literature Review” [tiab]**) AND (**"2015/05/01” [PDat]: "2021/02/26” [PDat]**)**

Dietary fiber search – 82 results

(“Dietary Fiber” [MeSH] OR “Dietary Fiber” [tiab] OR “Dietary Fibers” [tiab]) **AND** (“Cardiovascular Diseases” [MeSH] OR “Cardiovascular Disease” [tiab] OR “Cardiovascular Diseases” [tiab] OR “Heart Diseases” [MeSH] OR “Heart Diseases” [tiab] OR "myocardial infarction"[tiab] OR "myocardial infarctions"[tiab] OR "heart attack"[tiab] OR "heart attacks"[tiab] OR "sudden death"[tiab] OR "sudden deaths"[tiab] OR stroke[tiab] OR strokes[tiab] OR "cerebrovascular accident"[tiab] OR "cerebrovascular accidents"[tiab] OR “Diabetes Mellitus” [MeSH] OR “Diabetes Mellitus” [tiab] OR “Diabetes” [tiab] OR “Diabetes Mellitus, Type 2” [MeSH]) **AND (**“Meta-Analysis” [ptyp] OR “Meta-Analysis” [tiab] OR “Meta-analyses” [tiab] OR "Systematic Review" [tiab] OR "Systematic literature Review"[tiab] OR "Comprehensive Review"[tiab] OR "Comprehensive literature Review” [tiab]**) AND (**"2015/05/01” [PDat]: "2021/02/26” [PDat]**)**

Glycemic load/index search – 92 hits

(“Glycemic Load” [MeSH] OR “glycemic load” [tiab] OR “glycaemic load” [tiab] OR “glycemic index”[MeSH] OR “glycemic index”[tiab] OR “glycaemic index” [tiab]) **AND** (“Cardiovascular Diseases” [MeSH] OR “Cardiovascular Disease” [tiab] OR “Cardiovascular Diseases” [tiab] OR “Heart Diseases” [MeSH] OR “Heart Diseases” [tiab] OR "myocardial infarction"[tiab] OR "myocardial infarctions"[tiab] OR "heart attack"[tiab] OR "heart attacks"[tiab] OR "sudden death"[tiab] OR "sudden deaths"[tiab] OR stroke[tiab] OR strokes[tiab] OR "cerebrovascular accident"[tiab] OR "cerebrovascular accidents"[tiab] OR “Diabetes Mellitus” [MeSH] OR “Diabetes Mellitus” [tiab] OR “Diabetes” [tiab] OR “Diabetes Mellitus, Type 2” [MeSH]) **AND (**“Meta-Analysis” [ptyp] OR “Meta-Analysis” [tiab] OR “Meta-analyses” [tiab] OR "Systematic Review" [tiab] OR "Systematic literature Review"[tiab] OR "Comprehensive Review"[tiab] OR "Comprehensive literature Review” [tiab]**) AND (**"2015/05/01” [PDat]: "2021/02/26” [PDat]**)**

© 2022 Miller V et al. *JAMA Network Open.*

Dietary sodium search – 107 hits

("Sodium, Dietary" [MeSH] OR "Dietary Sodium" [tiab]) **AND** ("Cardiovascular Diseases" [MeSH] OR "Cardiovascular Disease" [tiab] OR "Cardiovascular Diseases" [tiab] OR "Heart Diseases" [MeSH] OR "Heart Diseases" [tiab] OR "myocardial infarction"[tiab] OR "myocardial infarctions"[tiab] OR "heart attack"[tiab] OR "heart attacks"[tiab] OR "sudden death"[tiab] OR "sudden deaths"[tiab] OR stroke[tiab] OR strokes[tiab] OR "cerebrovascular accident"[tiab] OR "cerebrovascular accidents"[tiab] OR "Diabetes Mellitus" [MeSH] OR "Diabetes Mellitus" [tiab] OR "Diabetes" [tiab] OR "Diabetes Mellitus, Type 2" [MeSH] OR "blood pressure" [MeSH] OR "blood pressure" [tiab] OR "hypertension" [MeSH] OR "hypertension" [tiab] OR "systolic blood pressure" [tiab] OR "diastolic blood pressure" [tiab]) **AND (**"Meta-Analysis" [ptyp] OR "Meta-Analysis" [tiab] OR "Meta-analyses" [tiab] OR "Systematic Review" [tiab] OR "Systematic literature Review"[tiab] OR "Comprehensive Review"[tiab] OR "Comprehensive literature Review" [tiab]**) AND (**"2015/05/01" [PDat]: "2021/02/26" [PDat]**)**

Dietary potassium search – 20 hits

("Potassium, Dietary" [MeSH] OR "Dietary Potassium" [tiab]) **AND** ("Cardiovascular Diseases" [MeSH] OR "Cardiovascular Disease" [tiab] OR "Cardiovascular Diseases" [tiab] OR "Heart Diseases" [MeSH] OR "Heart Diseases" [tiab] OR "myocardial infarction"[tiab] OR "myocardial infarctions"[tiab] OR "heart attack"[tiab] OR "heart attacks"[tiab] OR "sudden death"[tiab] OR "sudden deaths"[tiab] OR stroke[tiab] OR strokes[tiab] OR "cerebrovascular accident"[tiab] OR "cerebrovascular accidents"[tiab] OR "Diabetes Mellitus" [MeSH] OR "Diabetes Mellitus" [tiab] OR "Diabetes" [tiab] OR "Diabetes Mellitus, Type 2" [MeSH]) **AND (**"Meta-Analysis" [ptyp] OR "Meta-Analysis" [tiab] OR "Meta-analyses" [tiab] OR "Systematic Review" [tiab] OR "Systematic literature Review"[tiab] OR "Comprehensive Review"[tiab] OR "Comprehensive literature Review" [tiab]**) AND (**"2015/05/01" [PDat]: "2021/02/26" [PDat]**)**

Dietary calcium search – 42 hits

(("Calcium" [MeSH] AND "diet"[MeSH]) OR ("dietary" [tiab] AND "Calcium" [tiab])) **AND** ("Cardiovascular Diseases" [MeSH] OR "Cardiovascular Disease" [tiab] OR "Cardiovascular Diseases" [tiab] OR "Heart Diseases" [MeSH] OR "Heart Diseases" [tiab] OR "myocardial infarction"[tiab] OR "myocardial infarctions"[tiab] OR "heart attack"[tiab] OR "heart attacks"[tiab] OR "sudden death"[tiab] OR "sudden deaths"[tiab] OR stroke[tiab] OR strokes[tiab] OR "cerebrovascular accident"[tiab] OR "cerebrovascular accidents"[tiab] OR "Diabetes Mellitus" [MeSH] OR "Diabetes Mellitus" [tiab] OR "Diabetes" [tiab] OR "Diabetes

© 2022 Miller V et al. *JAMA Network Open.*

Mellitus, Type 2” [MeSH]) **AND (**“Meta-Analysis” [ptyp] OR “Meta-Analysis” [tiab] OR “Meta-analyses” [tiab] OR "Systematic Review" [tiab] OR "Systematic literature Review"[tiab] OR "Comprehensive Review"[tiab] OR "Comprehensive literature Review” [tiab]**) AND (**"2015/05/01” [PDat]: "2021/02/26” [PDat]**)**

Energy search – 16 hits

(“Energy Intake” [MeSH] OR “Energy Intake” [tiab] OR “Caloric Restriction” [MeSH] OR “Caloric Restriction” [tiab]) **AND** (“Cardiovascular Diseases” [MeSH] OR “Cardiovascular Disease” [tiab] OR “Cardiovascular Diseases” [tiab] OR “Heart Diseases” [MeSH] OR “Heart Diseases” [tiab] OR "myocardial infarction"[tiab] OR "myocardial infarctions"[tiab] OR "heart attack"[tiab] OR "heart attacks"[tiab] OR "sudden death"[tiab] OR "sudden deaths"[tiab] OR stroke[tiab] OR strokes[tiab] OR "cerebrovascular accident"[tiab] OR "cerebrovascular accidents"[tiab] OR “Diabetes Mellitus” [MeSH] OR “Diabetes Mellitus” [tiab] OR “Diabetes” [tiab] OR “Diabetes Mellitus, Type 2” [MeSH]) **AND (**“Meta-Analysis” [ptyp] OR “Meta-Analysis” [tiab] OR “Meta-analyses” [tiab] OR "Systematic Review" [tiab] OR "Systematic literature Review"[tiab] OR "Comprehensive Review"[tiab] OR "Comprehensive literature Review” [tiab]**) AND (**"2015/05/01” [PDat]: "2021/02/26” [PDat]**)**

© 2022 Miller V et al. *JAMA Network Open.*

eTable 1. Search Results, per Each Search Based on Types of Articles

**Risk Factor**	**Original search [2015]**	**Updated search [2021]**
**FOODS**		
Fruits	107	246
Fruit juices	9	37
Vegetables	94	184
Potatoes		12
Beans/legumes	17	56
Nuts/seeds	64	164
Whole grains	22	69
Refined grains		22
Dairy	56	166
Meats	50	101
Fish	92	118
Eggs	6	37
Sugar-sweetened beverages	108	347
Non-nutritive sweetened beverages		10
Coffee and tea	68	100
Cocoa	5	23
**NUTRIENTS**		
Dietary protein		99
Dietary fatty acids	448	763
Dietary cholesterol	5	14
Dietary fiber	46	73
Glycemic load/index	58	85
Dietary sodium	45	97
Dietary potassium	7	17
Dietary calcium	23	35
Energy	66	106
**Total**	**1396**	**2981**
**Total (excluding duplicates)**	**896**	**1867**

© 2022 Miller V et al. *JAMA Network Open.*

eFigure 1. Screening and Selection Process of Meta-analyses Evaluating Associations of Diet-Disease Relationships for Dietary Factors With Probable or Convincing Evidence on Cardiometabolic Diseases

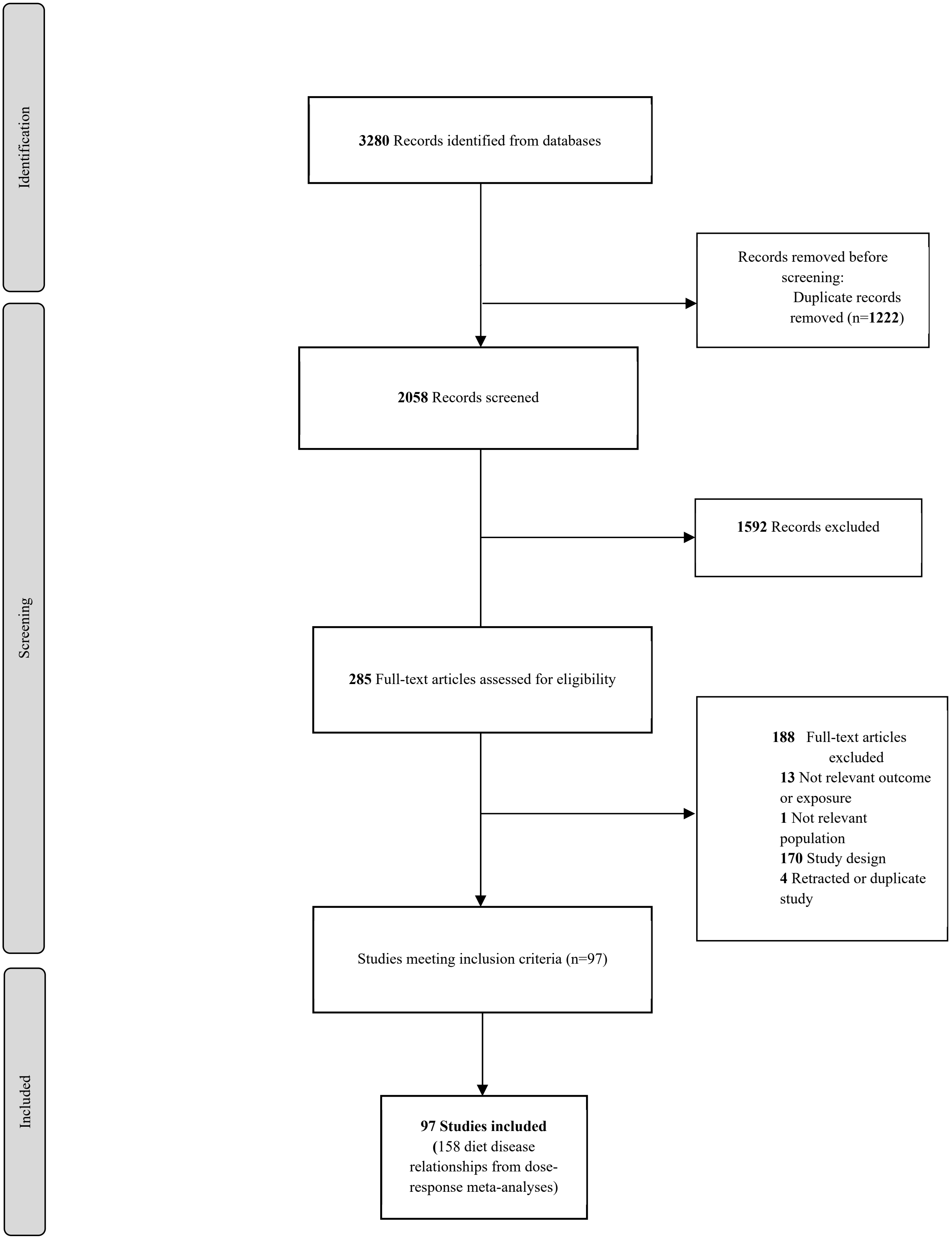

© 2022 Miller V et al. *JAMA Network Open.*

eFigure 2. Estimates of Etiologic Associations of Sodium and Systolic Blood Pressure

Source	Dietary Factor	Outcome	Studies in Each Meta−analysis	No. of Subjects	No. of Events	Unit	mm HG (95% CI)	I²
Mozaffarian 2014	Sodium	SBP, main effect, white, age 50, normotensives	103 RCTS (107 estimates)	6,970	N/A	per 2,300 mg/d (100 mmol/d)	3.74 (5.18, 2.29)	NR
Mozaffarian 2014	Sodium	SBP, additional effect per year of age < or > 50	103 RCTS (107 estimates)	6,970	N/A	per 2,300 mg/d (100 mmol/d)	0.105 (0.164, 0.047)	NR
Mozaffarian 2014	Sodium	SBP, additional effect among Blacks	103 RCTS (107 estimates)	6,970	N/A	per 2,300 mg/d (100 mmol/d)	2.49 (4.85, 0.13)	NR
Mozaffarian 2014	Sodium	SBP, additional effect among hypertensives	103 RCTS (107 estimates)	6,970	N/A	per 2,300 mg/d (100 mmol/d)	1.87 (3.63, 0.12)	NR

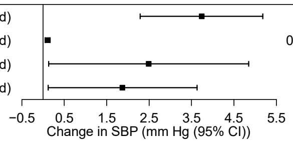

The boxes in the plot show the effect estimates from the meta-analyses and the horizontal lines through the boxes show the length of the confidence interval.
NR: Not reported
Dietary factors with probable or convincing evidence, based on the Bradford-Hill criteria, for associations on cardiometabolic (CMD) outcomes including cardiovascular disease (CVD), coronary heart disease (CHD), stroke, and type 2 diabetes. Diet-CMD relationships with <3 studies and papers that did not reference the individual studies included in the dose-response meta-analysis were not included.
Number of estimates can be higher than the number of studies if more than one arm in a randomized controlled trial, if estimates were separated by age or sex in prospective cohort studies, or more than one prospective cohort study was included in a single study.
Although we identified several meta-analyses on the relationship between dietary sodium intake, and SBP and DBP[7-11], no studies adjusted for age-, race-, and hypertension-status interactions which have been shown to mediate the association. For this relationship, we selected the study included in our previous review paper. Available evidence suggest that sodium increases mortality from CHD, stroke, and other-blood pressure related cardiovascular diseases through effects on SBP. For every year above or below age 50, there was 0.105 mm HG (95% CI: 0.047, 0.164) larger or smaller BP reduction, respectively. Effects on CVD vs. SBP were separately identified and are not independent (i.e., effects on CVD are at least partly mediated by SBP effects).

© 2022 Miller V et al. *JAMA Network Open.*

eTable 2. Estimates of Associations of Dietary Factors and Risk of Cardiometabolic Disease (Original Units and RRs [CIs])

Dietary Factor	Outcome	Studies in Each Meta-analysis	Source	No. of Subjects	No. of Events	Unit of RR	RR (95% CI)	Statistical Heterogeneity
**Foods and Beverages**								
Fruits	CVD	17 cohorts	Aune, 2017[12]	1,492,617	72,648	200 g/d	0.87 (0.82, 0.92)	$I^2 = 79.1\%$
	CHD	24 cohorts	Aune, 2017[12]	1,555,553	43,336	200 g/d	0.90 (0.86, 0.94)	$I^2 = 43.7\%$
	Stroke	16 cohorts	Aune, 2017[12]	964,142	46,203	200 g/d	0.82 (0.74, 0.90)	$I^2 = 72.9\%$
	Ischemic stroke	9 cohorts (10 estimates)	Aune, 2017[12]	412,875	11,577	200 g/d	0.78 (0.69, 0.89)	$I^2 = 57.5\%$
	Hemorrhagic stroke	7 cohorts	Aune, 2017[12]	655,406	6,728	200 g/d	0.66 (0.50, 0.86)	$I^2 = 56.9\%$
Vegetables	CVD	14 cohorts	Aune, 2017[12]	1,009,038	23,857	200 g/d	0.90 (0.87, 0.93)	$I^2 = 11.5\%$
	CHD	20 cohorts	Aune, 2017[12]	1,047,071	20,853	200 g/d	0.84 (0.79, 0.90)	$I^2 = 60.6\%$
	Stroke	13 cohorts	Aune, 2017[12]	441,670	14,973	200 g/d	0.87 (0.79, 0.96)	$I^2 = 63.4\%$
	Ischemic stroke	8 cohorts	Aune, 2017[12]	372,526	9,651	200 g/d	0.86 (0.76, 0.97)	$I^2 = 55.4\%$
Potatoes	Diabetes	6 cohorts (8 estimates)	Quan, 2020[13]	359,680	22,352	100 g/d	1.05 (1.02, 1.08)	I^2 = NR
Nuts/seeds	CVD	11 cohorts (12	Aune, 2016[14]	376,228	18,655	28 g/d	0.79 (0.70, 0.88)	$I^2 = 59.6\%$

Dietary Factor	Outcome	Studies in Each Meta-analysis	Source	No. of Subjects	No. of Events	Unit of RR	RR (95% CI)	Statistical Heterogeneity
		estimates)						
	CHD	10 cohorts (11 estimates)	Aune, 2016[14]	315,397	12,331	28 g/d	0.71 (0.63, 0.80)	$I^2 = 47.4\%$
Whole grains	CVD	9 cohorts (10 estimates)	Aune, 2016[15]	704,317	26,243	90 g/d	0.78 (0.73, 0.85)	$I^2 = 40\%$
	CHD	7 cohorts	Aune, 2016[15]	312,639	6,773	90 g/d	0.81 (0.75, 0.87)	$I^2 = 9\%$
	Ischemic stroke	3 cohorts	Chen, 2016[16]	114,773	NR	90 g/d	0.69 (0.55, 0.87)	I^2 = NR
	Diabetes	10 cohorts (12 estimates)	Schwingshackl, 2017[17]	459,603	22,267	30 g/d	0.87 (0.82, 0.93)	$I^2 = 91\%$
Red meats, unprocessed	CVD	4 cohorts	Zeraatkar, 2019[18]	65,736	NR	360 g/wk decrease	0.95 (0.85, 1.06)	$I^2 = 37.2\%$
	CHD	3 cohorts (4 estimates)	Bechthold, 2019[19]	151,373	6,659	100 g/d	1.15 (1.08, 1.23)	$I^2 = 0\%$
	Stroke	6 cohorts (7 estimates)	Bechthold, 2019[19]	341,767	17,900	100 g/d	1.12 (1.06, 1.17)	$I^2 = 0\%$
	Diabetes	11 cohorts	Zeraatkar, 2019[18]	531,843	NR	360 g/wk decrease	0.94 (0.89, 0.98)	$I^2 = 64.9\%$

© 2022 Miller V et al. *JAMA Network Open.*

Dietary Factor	Outcome	Studies in Each Meta-analysis	Source	No. of Subjects	No. of Events	Unit of RR	RR (95% CI)	Statistical Heterogeneity
Processed meat	CVD	3 cohorts	Zeraatkar, 2019[18]	200,421	NR	150 g/wk decrease	0.97 (0.87, 1.09)	I^2 = 59.2%
	CHD	3 cohorts	Bechthold, 2019[19]	151,373	6,659	50 g/d	1.27 (1.09, 1.49)	I^2 = 0%
	Stroke	6 cohorts	Zeraatkar, 2019[18]	254,742	13,113	150 g/wk decrease	0.94 (0.90, 0.98)	I^2 = 40.2%
	Ischemic stroke	5 cohorts	Bechthold, 2019[19]	NR	NR	50 g/d	1.12 (1.02, 1.23)	I^2 = 18%
	Diabetes	14 cohorts	Zeraatkar, 2019[18]	758,540	NR	150 g/wk decrease	0.85 (0.79, 0.92)	I^2 = 92.0%
Fish/seafood	CHD	15 cohorts	Bechthold, 2019[19]	479,657	14,056	100 g/d	0.88 (0.79, 0.99)	I^2 = 40%
	CHD in diabetes patients	3 cohorts	Jayedi, 2020[20]	8,464	NR	100 g/wk	0.92 (0.86, 0.98)	I^2 = 0.0%
	MI	11 cohorts	Jayedi, 2019[21]	398,221	8,468	15 g/d	0.96 (0.94, 0.99)	I^2 = 64.5%
	Stroke	14 cohorts (15 estimates)	Bechthold, 2019[19]	370,844	11,326	100 g/d	0.86 (0.75, 0.99)	I^2 = 25%
Yogurt	Diabetes	9 cohorts	Gijsbers, 2016[22]	438,140	36,125	50 g/d	0.94 (0.90, 0.97)	I^2 = 73.3%
Chocolate	CVD	12 cohorts (18 estimates)	Ren, 2019[23]	369,599	19,530	20 g/wk	0.982 (0.972, 0.992)	I^2 = 50.4%
	CHD	7 cohorts (8	Morze, 2020[24]	416,185	19,812	10 g/d	0.96 (0.93, 0.99)	I^2 = 29%

Dietary Factor	Outcome	Studies in Each Meta-analysis	Source	No. of Subjects	No. of Events	Unit of RR	RR (95% CI)	Statistical Heterogeneity
		estimates)						
	MI	4 cohorts	Larsson, 2016[25]	109,118	7,267	50 g/wk	0.95 (0.92, 0.98)	I^2 = 0%
	Stroke	7 cohorts	Morze, 2020[24]	275,070	9,087	10 g/d	0.90 (0.82, 0.98)	I^2 = 59%
	Hemorrhagic stroke	4 cohorts	Ren, 2019[23]	155,072	NR	20 g/wk	0.931 (0.871, 0.994)	I^2 = 0%
Milk	Stroke	15 cohorts (18 estimates)	Soedamah-Muthu, 2018[26]	4,381,604	25,377	200 g/d	0.92 (0.88, 0.97)	I^2 = 85.2%
Sugar-sweetened beverages	CVD	7 cohorts (10 estimates)	Yin, 2020[27]	198,388	16,999	250 mL/d	1.08 (1.02, 1.14)	I^2 = 43.0%
	CHD	4 cohorts	Yin, 2020[27]	173,753	7,407	250 mL/d	1.15 (1.09, 1.22)	I^2 = 0.0%
	Ischemic stroke	4 cohorts	Bechthold, 2019[19]	Not reported	NR	250 mL/d	1.07 (1.01, 1.15)	I^2 = 0%
	Diabetes	18 cohorts (19 estimates)	Qin, 2020[28]	1,010,392	34,788	250 mL/d	1.19 (1.13, 1.25)	I^2 = 82.4%
	↑ BMI (when baseline BMI <25 kg/m^2)	3 cohorts	Mozaffarian, 2011[29]	120,877	N/A	1 serving/d (8 oz/, 244g/d)	0.10 kg/m^2 (0.05, 0.15)	I^2 = NR
	↑ BMI (when baseline	3 cohorts	Mozaffarian, 2011[29]	120,877	N/A	1 serving/d (8 oz/, 244g/d)	0.23 kg/m^2 (0.14, 0.32)	I^2 = NR

© 2022 Miller V et al. *JAMA Network Open.*

Dietary Factor	Outcome	Studies in Each Meta-analysis	Source	No. of Subjects	No. of Events	Unit of RR	RR (95% CI)	Statistical Heterogeneity
	BMI ≥25 kg/m^2)							
Tea	Stroke	11 cohorts (13 estimates)	Chung, 2020[30]	455,378	NR	1 cup/d (236.6 mL/d)	0.96 (0.93, 0.99)	I^2 = 63.9%
**Nutrients**								
Dietary fiber	CVD	8 cohorts	Reynolds, 2019[31]	1.8 million-person-years	NR	8 g/d	0.78 (0.68, 0.90)	I^2 = 79%
	CHD	9 cohorts	Reynolds, 2019[31]	2.5 million-person-years	NR	8 g/d	0.81 (0.73, 0.90)	I^2 = 74%
	Stroke	11 cohorts	Reynolds, 2019[31]	4.5 million-person-years	NR	8 g/d	0.90 (0.85, 0.95)	I^2 = 13%
	Diabetes	15 cohorts	InterAct, 2015[32]	414,711	26,131	10 g/d	0.91 (0.87, 0.96)	I^2 = 29.4%
Cereal fiber	Diabetes	12 cohorts	InterAct, 2015[32]	452,367	27,512	10 g/d	0.75 (0.65, 0.86)	I^2 = 75.1%
Fruit fiber	Stroke	3 cohorts	Reynolds, 2019[31]	2.2 million-person-years	NR	2 g/d	0.95 (0.92, 0.98)	I^2 = 4%
Vegetable fiber	Stroke	3 cohorts	Reynolds, 2019[31]	2.2 million-person-years	NR	2 g/d	0.91 (0.88, 0.95)	I^2 = 2%
Glycemic index	CHD	8 cohorts (10 estimates)	Livesey, 2019[33]	319,288	NR	10 units	1.24 (1.12, 1.38)	I^2 = 10%
	Diabetes	9 cohorts (10	Livesey, 2019[34]	346,465	18,063	10 units	1.27 (1.15, 1.40)	I^2 = 68%

© 2022 Miller V et al. *JAMA Network Open.*

Dietary Factor	Outcome	Studies in Each Meta-analysis	Source	No. of Subjects	No. of Events	Unit of RR	RR (95% CI)	Statistical Heterogeneity
		estimates)						
Glycemic load	CHD	8 cohorts (11 estimates)	Livesey, 2019[33]	301,589	NR	65 g/d /2000kcal	1.44 (1.25, 1.65)	I^2 = 18%
	Diabetes	12 studies (15 cohorts)	Livesey, 2019[34]	624,614	25,370	80 g/d/2000 kcal	1.26 (1.15, 1.37)	I^2 = 35%
PUFA replacing CHO	CHD	9 cohorts (12 estimates)	Farvid, 2014[35]	262,612	12,198	per 5% E/d	0.90 (0.85, 0.94)	I^2 = 47.3%
	Diabetes	9 cohorts (12 estimates)	Merino, 2019[36]	81,810	20,015	per 5% E/d	0.90 (0.82, 0.98)	I^2 = 18%
PUFA replacing SFA	CHD	8 cohorts (11 estimates)	Farvid, 2014[35]	262,612	12,198	per 5% E/d	0.91 (0.87, 0.96)	I^2 = 55.9%
Trans-fats	CHD	4 cohorts	Mozaffarian, 2006[37]	139,836	4,965	per 2% E/d	1.23 (1.11, 1.37)	I^2 = NR
Total protein	Diabetes	7 cohorts (10 estimates)	Zhao, 2019[38]	403,109	32,663	per 5% E/d	1.09 (1.04, 1.13)	I^2 = 42.0%
Animal protein	Diabetes	6 cohorts (9 estimates)	Zhao, 2019[38]	357,893	30,591	per 5% E/d	1.12 (1.08, 1.17)	I^2 = 14.0%
Sodium	Stroke	14 cohorts	Jayedi, 2019[39]	253,449	9,877	1 g/d	1.06 (1.02, 1.10)	I^2 = 60.4%

© 2022 Miller V et al. *JAMA Network Open.*

Dietary Factor	Outcome	Studies in Each Meta-analysis	Source	No. of Subjects	No. of Events	Unit of RR	RR (95% CI)	Statistical Heterogeneity
	SBP, main effect, white, age 50, normotensives	103 RCTS (107 estimates)	Mozaffarian 2014[40]	6,970	N/A	Per 2,300 mg/d (100 mmol/d)	3.74 (5.18, 2.29)	I^2 = NR
	SBP, additional effect per year of age < or > 50	103 RCTS (107 estimates)	Mozaffarian 2014[40]	6,970	N/A	Per 2,300 mg/d (100 mmol/d)	0.105 (0.164, 0.047)	I^2 = NR
	SBP, additional effect among Blacks	103 RCTS (107 estimates)	Mozaffarian 2014[40]	6,970	N/A	Per 2,300 mg/d (100 mmol/d)	2.49 (4.85, 0.13)	I^2 = NR
	SBP, additional effect among hypertensives	103 RCTS (107 estimates)	Mozaffarian 2014[40]	6,970	N/A	Per 2,300 mg/d (100 mmol/d)	1.87 (3.63, 0.12)	I^2 = NR
Potassium	Stroke	9 cohorts (11 estimates)	D'Elia, 2011[41]	233,606	7,066	Per 1,000 mg/d (25.7 mmol/d)	0.87 (0.79, 0.94)	I^2 = 55%

Effect sizes (RR and 95% CI) and unit of RR as reported by the published meta-analysis.
PUFA: polyunsaturated fats; CHO: carbohydrates; SFA: saturated fat; %E/d: percent energy per day.
NR: Not reported
Dietary factors with probable or convincing evidence, based on the Bradford-Hill criteria for assessing causality, for associations on cardiometabolic (CMD) outcomes including cardiovascular disease (CVD), coronary heart disease (CHD), stroke, and type 2 diabetes. Diet-CMD relationships with <3 studies and papers that did not reference the individual studies included in the dose-response meta-analysis were not included.
Fruits- exclude 100% juices, and vegetables exclude vegetable juices, starchy vegetables such as potatoes and corn, and salted or pickled vegetables. Because individual studies may include potatoes in the vegetable category, the associations identified for vegetables should be considered as representing the outcome of vegetables, including potatoes. Associations of potatoes were also evaluated separately.
Evidence suggests that SSBs are associated with increased risk because they affect both BMI and BMI-independent factors of type 2 diabetes and cardiovascular outcomes. Several meta-analyses found an association between SSB intake and incident overweight or obesity, but none reported on the association between changes in SSB intake and weight gain. For this association, we selected a pooled analysis of 3 prospective cohort studies[29].

© 2022 Miller V et al. *JAMA Network Open.*

Glycemic load is calculated as the glycemic index of a food multiplied by its carbohydrate content. Higher values reflect both higher glycemic index and higher quantities of refines grains, starches, and sugars. Evidence of associations of dietary fiber was also identified. Glycemic load and dietary fiber overlap with foods in Figure 2, including fruits, vegetables, potatoes, beans or legumes, nuts or seeds, whole grains, and refined grains. Although the Reynolds et al[31] meta-analysis contained 1 additional primary study than Livesey et al[34], we did not select it because it included several primary studies with poor dietary instrument validity that resulted in a null association between glycemic index and diabetes.

Sodium was assessed by 24-hr dietary recall, food frequency questionnaire, or 24-hr urine excretion.

No meta-analyses were identified for potassium. For this association, the study identified in the previous review[6] was selected.

© 2022 Miller V et al. *JAMA Network Open.*

eTable 3. Reasons for Excluding Dietary Factor-CMD Relationships

**Dietary Factor**	**Outcome**	**Reason(s) for Exclusion**
Beans/legumes[42]	CVD	Consistency could not be assessed because the meta-analysis did not reference the primary studies or include a forest plot
Nuts/seeds[43]	Hemorrhagic stroke	Consistency could not be assessed because the meta-analysis did not reference the primary studies or include a forest plot
Fruit juice[44]	CVD	<3 primary studies included in the meta-analysis
	Stroke	<3 primary studies included in the meta-analysis
SSBs[45]	MI	<3 primary studies included in the meta-analysis
Non-nutritive sweetened beverages[27,28]	CVD	Lack of biological plausibility, and supportive experiment from RCTs and cohorts of risk factors
	CHD	Lack of biological plausibility, and supportive experiment from RCTs and cohorts of risk factors
	Stroke	Lack of biological plausibility, and supportive experiment from RCTs and cohorts of risk factors
	Diabetes	Lack of supportive experiment, including from RCTs and cohorts of risk factors
Vegetable fiber[31]	CHD	<3 primary studies included in the meta-analysis
PUFA[46]	CVD	Lack of biological plausibility, and supportive experiment from RCTs and cohorts of risk factors
PUFA replacing SFA[47]	CVD in diabetes patients	<3 primary studies included in the meta-analysis
MUFA replacing CHO[36]	Diabetes	Lack of biological plausibility, and supportive experiment from RCTs and cohorts of risk factors
Coffee[48]	Diabetes	Lack of supportive experiment, including from RCTs and cohorts of risk factors
SFA[49,50]	CVD	Included trials were mostly PUFA replacing SFA
	Stroke	Lack of biological plausibility
	Hemorrhagic stroke	Consistency could not be assessed because the meta-analysis did not reference the primary studies or include a forest plot
Sodium[51]	CVD	Consistency could not be assessed because the meta-analysis did not reference the primary studies or include a forest plot

Although meta-analyses were available for several diet-CMD relationships, no significant associations were found, such as eggs[52,53], SFA[36,46,54], milk[22,26,55,56], legumes[17,19,42], refined grains[15,17,19], nuts[14,17,43], MUFA[36,46,47,54], cheese[22,55,56], and yogurt[55,56].

Our updated search did not identify dose-response meta-analyses on lean fish, fatty fish, seafood omega-3, plant omega-3, dietary cholesterol, dietary calcium, or total energy.

© 2022 Miller V et al. *JAMA Network Open.*

eReferences

1 Hill, A. B. The Environment and Disease: Association or Causation? *Proc R Soc Med* **58**, 295-300 (1965).

2 WHO. The World Health Report 2002: Reducing Risks, Promoting Healthy Life. (World Health Organization, 2002).

3 World Cancer Research Fund/ American Institute for Cancer Research. Food, Nutrition, Physical Activity, and the Prevention of Cancer: a Global Perspective. (Washington DC: AICR, 2007).

4 World Cancer Research Fund/ American Institute for Cancer Research. *Continuous Update Project (CUP)*, <http://www.dietandcancerreport.org/cup/report_overview/index.php> (

5 Mozaffarian, D. Dietary and Policy Priorities for Cardiovascular Disease, Diabetes, and Obesity: A Comprehensive Review. *Circulation* **133**, 187-225, doi:10.1161/circulationaha.115.018585 (2016).

6 Micha, R. *et al.* Etiologic effects and optimal intakes of foods and nutrients for risk of cardiovascular diseases and diabetes: Systematic reviews and meta-analyses from the Nutrition and Chronic Diseases Expert Group (NutriCoDE). *PLoS One* **12**, e0175149, doi:10.1371/journal.pone.0175149 (2017).

7 Graudal, N., Hubeck-Graudal, T., Jürgens, G. & Taylor, R. S. Dose-response relation between dietary sodium and blood pressure: a meta-regression analysis of 133 randomized controlled trials. *Am J Clin Nutr* **109**, 1273-1278, doi:10.1093/ajcn/nqy384 (2019).

8 Graudal, N. A., Hubeck-Graudal, T. & Jurgens, G. Effects of low sodium diet versus high sodium diet on blood pressure, renin, aldosterone, catecholamines, cholesterol, and triglyceride. *Cochrane Database Syst Rev* **4**, Cd004022, doi:10.1002/14651858.CD004022.pub4 (2017).

9 Graudal, N. A., Hubeck-Graudal, T. & Jurgens, G. Effects of low sodium diet versus high sodium diet on blood pressure, renin, aldosterone, catecholamines, cholesterol, and triglyceride. *Cochrane Database Syst Rev* **12**, Cd004022, doi:10.1002/14651858.CD004022.pub5 (2020).

10 Gay, H. C., Rao, S. G., Vaccarino, V. & Ali, M. K. Effects of Different Dietary Interventions on Blood Pressure: Systematic Review and Meta-Analysis of Randomized Controlled Trials. *Hypertension* **67**, 733-739, doi:10.1161/hypertensionaha.115.06853 (2016).

11 Huang, L. *et al.* Effect of dose and duration of reduction in dietary sodium on blood pressure levels: systematic review and meta-analysis of randomised trials. *Bmj* **368**, m315, doi:10.1136/bmj.m315 (2020).

12 Aune, D. *et al.* Fruit and vegetable intake and the risk of cardiovascular disease, total cancer and all-cause mortality-a systematic review and dose-response meta-analysis of prospective studies. *Int J Epidemiol* **46**, 1029-1056, doi:10.1093/ije/dyw319 (2017).

13 Quan, W. *et al.* Processed potatoes intake and risk of type 2 diabetes: a systematic review and meta-analysis of nine prospective cohort studies. *Crit Rev Food Sci Nutr*, 1-9, doi:10.1080/10408398.2020.1843395 (2020).

14 Aune, D. *et al.* Nut consumption and risk of cardiovascular disease, total cancer, all-cause and cause-specific mortality: a systematic review and dose-response meta-analysis of prospective studies. *BMC Med* **14**, 207, doi:10.1186/s12916-016-0730-3 (2016).

15 Aune, D. *et al.* Whole grain consumption and risk of cardiovascular disease, cancer, and all cause and cause specific mortality: systematic review and dose-response meta-analysis of prospective studies. *Bmj* **353**, i2716, doi:10.1136/bmj.i2716 (2016).

16 Chen, J. *et al.* Meta-Analysis of the Association Between Whole and Refined Grain Consumption and Stroke Risk Based on Prospective Cohort Studies. *Asia Pac J Public Health* **28**, 563-575, doi:10.1177/1010539516650722 (2016).

17 Schwingshackl, L. *et al.* Food groups and risk of type 2 diabetes mellitus: a systematic review and meta-analysis of prospective studies. *Eur J Epidemiol* **32**, 363-375, doi:10.1007/s10654-017-0246-y (2017).

18 Zeraatkar, D. *et al.* Red and Processed Meat Consumption and Risk for All-Cause Mortality and Cardiometabolic Outcomes: A Systematic Review and Meta-analysis of Cohort Studies. *Ann Intern Med* **171**, 703-710, doi:10.7326/m19-0655 (2019).

19 Bechthold, A. *et al.* Food groups and risk of coronary heart disease, stroke and heart failure: A systematic review and dose-response meta-analysis of prospective studies. *Crit Rev Food Sci Nutr* **59**, 1071-1090, doi:10.1080/10408398.2017.1392288 (2019).

© 2022 Miller V et al. *JAMA Network Open.*

20 Jayedi, A., Soltani, S., Abdolshahi, A. & Shab-Bidar, S. Fish consumption and the risk of cardiovascular disease and mortality in patients with type 2 diabetes: a dose-response meta-analysis of prospective cohort studies. *Crit Rev Food Sci Nutr*, 1-11, doi:10.1080/10408398.2020.1764486 (2020).
21 Jayedi, A., Zargar, M. S. & Shab-Bidar, S. Fish consumption and risk of myocardial infarction: a systematic review and dose-response meta-analysis suggests a regional difference. *Nutr Res* **62**, 1-12, doi:10.1016/j.nutres.2018.10.009 (2019).
22 Gijsbers, L. *et al.* Consumption of dairy foods and diabetes incidence: a dose-response meta-analysis of observational studies. *Am J Clin Nutr* **103**, 1111-1124, doi:10.3945/ajcn.115.123216 (2016).
23 Ren, Y. *et al.* Chocolate consumption and risk of cardiovascular diseases: a meta-analysis of prospective studies. *Heart* **105**, 49-55, doi:10.1136/heartjnl-2018-313131 (2019).
24 Morze, J. *et al.* Chocolate and risk of chronic disease: a systematic review and dose-response meta-analysis. *Eur J Nutr* **59**, 389-397, doi:10.1007/s00394-019-01914-9 (2020).
25 Larsson, S. C., Åkesson, A., Gigante, B. & Wolk, A. Chocolate consumption and risk of myocardial infarction: a prospective study and meta-analysis. *Heart* **102**, 1017-1022, doi:10.1136/heartjnl-2015-309203 (2016).
26 Soedamah-Muthu, S. S. & de Goede, J. Dairy Consumption and Cardiometabolic Diseases: Systematic Review and Updated Meta-Analyses of Prospective Cohort Studies. *Current nutrition reports* **7**, 171-182, doi:10.1007/s13668-018-0253-y (2018).
27 Yin, J. *et al.* Intake of Sugar-Sweetened and Low-Calorie Sweetened Beverages and Risk of Cardiovascular Disease: A Meta-Analysis and Systematic Review. *Adv Nutr*, doi:10.1093/advances/nmaa084 (2020).
28 Qin, P. *et al.* Sugar and artificially sweetened beverages and risk of obesity, type 2 diabetes mellitus, hypertension, and all-cause mortality: a dose-response meta-analysis of prospective cohort studies. *Eur J Epidemiol* **35**, 655-671, doi:10.1007/s10654-020-00655-y (2020).
29 Mozaffarian, D., Hao, T., Rimm, E. B., Willett, W. C. & Hu, F. B. Changes in diet and lifestyle and long-term weight gain in women and men. *N Engl J Med* **364**, 2392-2404, doi:10.1056/NEJMoa1014296 (2011).
30 Chung, M. *et al.* Dose-Response Relation between Tea Consumption and Risk of Cardiovascular Disease and All-Cause Mortality: A Systematic Review and Meta-Analysis of Population-Based Studies. *Adv Nutr* **11**, 790-814, doi:10.1093/advances/nmaa010 (2020).
31 Reynolds, A. *et al.* Carbohydrate quality and human health: a series of systematic reviews and meta-analyses. *Lancet* **393**, 434-445, doi:10.1016/S0140-6736(18)31809-9 (2019).
32 Dietary fibre and incidence of type 2 diabetes in eight European countries: the EPIC-InterAct Study and a meta-analysis of prospective studies. *Diabetologia* **58**, 1394-1408, doi:10.1007/s00125-015-3585-9 (2015).
33 Livesey, G. & Livesey, H. Coronary Heart Disease and Dietary Carbohydrate, Glycemic Index, and Glycemic Load: Dose-Response Meta-analyses of Prospective Cohort Studies. *Mayo Clin Proc Innov Qual Outcomes* **3**, 52-69, doi:10.1016/j.mayocpiqo.2018.12.007 (2019).
34 Livesey, G. *et al.* Dietary Glycemic Index and Load and the Risk of Type 2 Diabetes: A Systematic Review and Updated Meta-Analyses of Prospective Cohort Studies. *Nutrients* **11**, doi:10.3390/nu11061280 (2019).
35 Farvid, M. S. *et al.* Dietary linoleic acid and risk of coronary heart disease: a systematic review and meta-analysis of prospective cohort studies. *Circulation* **130**, 1568-1578, doi:10.1161/circulationaha.114.010236 (2014).
36 Merino, J. *et al.* Quality of dietary fat and genetic risk of type 2 diabetes: individual participant data meta-analysis. *Bmj* **366**, l4292, doi:10.1136/bmj.l4292 (2019).
37 Mozaffarian, D., Katan, M. B., Ascherio, A., Stampfer, M. J. & Willett, W. C. Trans fatty acids and cardiovascular disease. *N Engl J Med* **354**, 1601-1613, doi:10.1056/NEJMra054035 (2006).
38 Zhao, L. G. *et al.* Dietary protein intake and risk of type 2 diabetes: a dose-response meta-analysis of prospective studies. *Eur J Nutr* **58**, 1351-1367, doi:10.1007/s00394-018-1737-7 (2019).
39 Jayedi, A., Ghomashi, F., Zargar, M. S. & Shab-Bidar, S. Dietary sodium, sodium-to-potassium ratio, and risk of stroke: A systematic review and nonlinear dose-response meta-analysis. *Clin Nutr* **38**, 1092-1100, doi:10.1016/j.clnu.2018.05.017 (2019).

© 2022 Miller V et al. *JAMA Network Open.*

40 Mozaffarian, D. *et al.* Global sodium consumption and death from cardiovascular causes. *N Engl J Med* **371**, 624-634, doi:10.1056/NEJMoa1304127 (2014).
41 D'Elia, L., Barba, G., Cappuccio, F. P. & Strazzullo, P. Potassium intake, stroke, and cardiovascular disease a meta-analysis of prospective studies. *J Am Coll Cardiol* **57**, 1210-1219, doi:10.1016/j.jacc.2010.09.070 (2011).
42 Viguiliouk, E. *et al.* Associations between Dietary Pulses Alone or with Other Legumes and Cardiometabolic Disease Outcomes: An Umbrella Review and Updated Systematic Review and Meta-analysis of Prospective Cohort Studies. *Adv Nutr* **10**, S308-s319, doi:10.1093/advances/nmz113 (2019).
43 Becerra-Tomás, N. *et al.* Nut consumption and incidence of cardiovascular diseases and cardiovascular disease mortality: a meta-analysis of prospective cohort studies. *Nutr Rev* **77**, 691-709, doi:10.1093/nutrit/nuz042 (2019).
44 D'Elia, L., Dinu, M., Sofi, F., Volpe, M. & Strazzullo, P. 100% Fruit juice intake and cardiovascular risk: a systematic review and meta-analysis of prospective and randomised controlled studies. *Eur J Nutr*, doi:10.1007/s00394-020-02426-7 (2020).
45 Narain, A., Kwok, C. S. & Mamas, M. A. Soft drinks and sweetened beverages and the risk of cardiovascular disease and mortality: a systematic review and meta-analysis. *Int J Clin Pract* **70**, 791-805, doi:10.1111/ijcp.12841 (2016).
46 Zhu, Y., Bo, Y. & Liu, Y. Dietary total fat, fatty acids intake, and risk of cardiovascular disease: a dose-response meta-analysis of cohort studies. *Lipids Health Dis* **18**, 91, doi:10.1186/s12944-019-1035-2 (2019).
47 Schwab, U., Reynolds, A. N., Sallinen, T., Rivellese, A. A. & Risérus, U. Dietary fat intakes and cardiovascular disease risk in adults with type 2 diabetes: a systematic review and meta-analysis. *Eur J Nutr*, doi:10.1007/s00394-021-02507-1 (2021).
48 Carlström, M. & Larsson, S. C. Coffee consumption and reduced risk of developing type 2 diabetes: a systematic review with meta-analysis. *Nutr Rev* **76**, 395-417, doi:10.1093/nutrit/nuy014 (2018).
49 Kang, Z. Q., Yang, Y. & Xiao, B. Dietary saturated fat intake and risk of stroke: Systematic review and dose-response meta-analysis of prospective cohort studies. *Nutr Metab Cardiovasc Dis* **30**, 179-189, doi:10.1016/j.numecd.2019.09.028 (2020).
50 Hooper, L. *et al.* Reduction in saturated fat intake for cardiovascular disease. *Cochrane Database Syst Rev* **5**, Cd011737, doi:10.1002/14651858.CD011737.pub2 (2020).
51 Wang, Y. J., Yeh, T. L., Shih, M. C., Tu, Y. K. & Chien, K. L. Dietary Sodium Intake and Risk of Cardiovascular Disease: A Systematic Review and Dose-Response Meta-Analysis. *Nutrients* **12**, doi:10.3390/nu12102934 (2020).
52 Drouin-Chartier, J. P. *et al.* Egg consumption and risk of cardiovascular disease: three large prospective US cohort studies, systematic review, and updated meta-analysis. *BMJ* **368**, m513, doi:10.1136/bmj.m513 (2020).
53 Godos, J. *et al.* Egg consumption and cardiovascular risk: a dose-response meta-analysis of prospective cohort studies. *Eur J Nutr*, doi:10.1007/s00394-020-02345-7 (2020).
54 Neuenschwander, M. *et al.* Intake of dietary fats and fatty acids and the incidence of type 2 diabetes: A systematic review and dose-response meta-analysis of prospective observational studies. *PLoS Med* **17**, e1003347, doi:10.1371/journal.pmed.1003347 (2020).
55 de Goede, J., Soedamah-Muthu, S. S., Pan, A., Gijsbers, L. & Geleijnse, J. M. Dairy Consumption and Risk of Stroke: A Systematic Review and Updated Dose-Response Meta-Analysis of Prospective Cohort Studies. *J Am Heart Assoc* **5**, doi:10.1161/jaha.115.002787 (2016).
56 Guo, J. *et al.* Milk and dairy consumption and risk of cardiovascular diseases and all-cause mortality: dose-response meta-analysis of prospective cohort studies. *Eur J Epidemiol* **32**, 269-287, doi:10.1007/s10654-017-0243-1 (2017).

© 2022 Miller V et al. *JAMA Network Open.*
